# Error Performance of Amplitude Shift Keying-Type Asymmetric Quantum Communication Systems

**DOI:** 10.3390/e24050708

**Published:** 2022-05-16

**Authors:** Tiancheng Wang, Tsuyoshi Sasaki Usuda

**Affiliations:** 1 Faculty of Engineering, Kanagawa University, Yokohama 221-8686, Kanagawa, Japan; 2 Graduate School of Information Science and Technology, Aichi Prefectural University, Nagakute 480-1198, Aichi, Japan

**Keywords:** entanglement, quasi-Bell state, asymmetric communication system, error performance

## Abstract

We propose an amplitude shift keying-type asymmetric quantum communication (AQC) system that uses an entangled state. As a first step toward development of this system, we evaluated and considered the communication performance of the proposed receiver when applied to the AQC system using a two-mode squeezed vacuum state (TSVS), the maximum quasi-Bell state, and the non-maximum quasi-Bell state, along with an asymmetric classical communication (ACC) system using the coherent state. Specifically, we derived an analytical expression for the error probability of the AQC system using the quasi-Bell state. Comparison of the error probabilities of the ACC system and the AQC systems when using the TSVS and the quasi-Bell state shows that the AQC system using the quasi-Bell state offers a clear performance advantage under specific conditions. Additionally, it was clarified that there are cases where the universal lower bound on the error probability for the AQC system was almost achieved when using the quasi-Bell state, unlike the case in which the TSVS was used.

## 1. Introduction

Entanglement [1] is an important phenomenon for quantum protocols. Entanglement is a nonlocal correlation that works with multiple quantum systems. This correlation can be maintained regardless of the distance between these multiple quantum systems. The quantum cryptographic protocol called E91 [2], quantum superdense coding [3], and quantum teleportation [4], which were all proposed in the 1990s, are well known as quantum communication protocols that apply entanglement. In addition, quantum illumination [5] and quantum reading [6], which are quantum metrology protocols based on entanglement and were proposed around 2010, have also been attracting increasing attention in recent years.

Many of the quantum communication protocols described above that use entanglement belong to the class of symmetric communication systems. Symmetric communication systems have the same transmission capability, regardless of the direction of communication. In contrast, asymmetric communication systems have different transmission capabilities that depend on the direction of communication (e.g., [7,8]). The differences in transmission capability in this case are caused by the differences between the physical resources that can be used on the two sides of the communication process. However, as far as we know, there are no asymmetric communication systems which essentially utilize quantum mechanical phenomena such as entanglement. With this in mind, we define the following: asymmetric systems using quantum and classical communication protocols are called asymmetric quantum communication (AQC) systems and asymmetric classical communication (ACC) systems, respectively. In this paper, we consider the quantum communication protocols with entanglement. Typical examples of ACC systems include terrestrial-to-satellite communications, communication between a mobile device and a cellular base station, and communication between an Internet of Things (IoT) device and an IoT base station. For example, in an IoT-based ACC system, there is a major difference between the transmission capabilities of a small battery-driven IoT device—where the battery is replaced once every few years and the microprocessor unit can only perform simple calculation processes—and that of a base station with an abundant power supply and high processing capacity. In this work, the side with the low transmission capability, e.g., satellites, mobile phones, and IoT devices, is called Alice, and the side with the high transmission capability, e.g., ground base stations, mobile phone base stations, and IoT base stations, is called Bob. Taking an IoT-based ACC system as an example, the usage scenario of that is considered as a simple model in this paper follows: (1) Bob (the IoT base station) tells Alice (the IoT device) whether to sense the physical environment; (2) if yes, Alice senses the physical environment and transmits the corresponding data to Bob. In general, information leakage is prone to occur in the channel of (2), and the system must perform some communication protocols, such as lightweight cryptography. One of the security problems for the asymmetric communication system can be described as follows: the low processing capacity struggles to always provide a high security level for the data transmitted from such an IoT device, because the frequency of upgrading the device may be low and the development of code breaking technique is fast. We aimed to develop a new AQC system to improve the reliability and security of communications from Alice to Bob, and also to reduce Alice’s energy costs by introducing entanglement into the asymmetric communication system. This paper represents the first step in this research.

To develop the required AQC system, it will be necessary to clarify the effects of deterioration of the quantum effects in the various channels with respect to the entangled state. Reference [9] dealt with the quantum channel discrimination problem using beam splitters with reflectivities of $R_{0}$ and $R_{1}\left(0\leq R_{0}<R_{1}\leq 1\right)$. In this channel model, a quantum state source (i.e., a light source) produces an entangled state, and these two modes are labeled S (signal mode: mode S) and A (ancilla mode: mode A). Light corresponding to mode S is directed toward one beam splitter with a reflectivity of either $R_{0}$ or $R_{1}$; the subsequently reflected light is then collected using a detector. The other beam, which corresponds to mode A, is sent to the detector directly. The detector then distinguishes the two channels that correspond to $R_{0}$ and $R_{1}$ by performing optimum quantum measurements (i.e., joint measurements) of the two light beams. In reference [9], the Einstein–Podolsky–Rosen (EPR) state, which consists of the *m*-fold tensor product of the two-mode squeezed vacuum state (TSVS) [10], was used as the light source, and the performance with regard to the error probability when using the EPR state was evaluated using its upper bound, which is defined by the Chernoff bound [11], and the lower bound, which is defined by the fidelity. As a result, it was found that the lower bound on the error probability when using the EPR state may almost reach the universal lower bound. In fact, if we consider that the communication system is used in such a manner that Alice operates the two beam splitters to transmit binary information to Bob based on differences in reflectivity (i.e., the differences in the amplitudes of the reflected light beams when subjected to different energy attenuation levels), the model in reference [9] would be an amplitude shift keying (ASK)-type AQC system. Therefore, it can be said that the work in this paper uses the same model for a different purpose to that of reference [9].

When considering aspects of the communication performance, it is necessary to perform instantaneous performance evaluations, but not using the Chernoff bound that corresponds to the case in which *m*-shot optical pulses are applied; instead, the error probability when a one-shot optical pulse is used here. In this case, the TSVS, i.e., the EPR state when m=1, is considered, rather than the EPR state. In addition, there is a quasi-Bell state [12] that is constructed using nonorthogonal quantum states such as coherent states, but it becomes the maximum entangled state (maximum quasi-Bell state). It has been shown that the attenuation resistance of this state is strong, depending on application protocols, and it has been studied actively (e.g., [13,14]) since the publication of reference [12]. In addition, study of the application of a quasi-Bell state that is not the maximum entangled state (i.e., a non-maximum quasi-Bell state) has advanced, and it has been reported that the non-maximum quasi-Bell state is superior to the maximum quasi-Bell state for use in certain protocols, such as quantum teleportation [15]. In particular, it was recently clarified that the quasi-Bell state is superior to both the TSVS and conventional laser radar in terms of its performance for quantum illumination with attenuation (i.e., the model used in this paper with $R_{0}=0$) [16,17,18,19].

Based on the discussion above, we aim to clarify Bob’s communication performance based on the error probability criterion as a first step toward development of an ASK-type AQC system (hereinafter referred to simply as the AQC system). Specifically, by using the Schrödinger picture to describe the time evolution of both the quasi-Bell state and the TSVS, we evaluate and compare the error probabilities that occur when using these states, and thus consider the basic characteristics, i.e., the error performance, of the AQC system. We also compare these results with the error probability characteristics of an ACC system which was constructed using a coherent state source and an optimum classical measurement approach, along with the universal lower bound on the error probability when the use of any multimode quantum state is allowed. As the main results of this analysis, we derive an analytical expression for the error probability of the AQC system when using the quasi-Bell state. Then, by investigating the numerical characteristics of the system using this analytical expression with various reflectivities and the average number of photons, we demonstrate that the AQC system using the quasi-Bell state is not only always superior to the ACC system, but also is asymptotically superior to the same AQC system using the TSVS. Additionally, in contrast to reference [9], which shows that using the EPR state asymptotically outperforms using the coherent state, this paper shows that the ACC system asymptotically outperforms the AQC system when using the TSVS. Finally, we show that the AQC system using the quasi-Bell state may almost reach the universal lower bound on the error probability, unlike the AQC system using the TSVS.

The rest of this paper is organized as follows. Section 2 describes the TSVS, the quasi-Bell state, and the ACC system. Section 3 describes the model of the AQC system when using the entangled states, and also provides a description of Bob’s received quantum states in the AQC system. Section 4 presents an analytical expression for the error probability of the AQC system when using the quasi-Bell state. In Section 5, by using the analytical expression obtained in Section 4, the system error performance is given and is compared numerically with the error probabilities of both the ACC system and the AQC system when using the TSVS and the universal lower bound on the error probability.

## 2. Basic Theory

### 2.1. Quantum State

The state of a quantum system (i.e., the quantum state ρ) is expressed using a density operator. This density operator is a nonnegative Hermitian operator on Hilbert space and satisfies

(1) $$\begin{array}{c} \rho \geq 0,\;\mathrm{Tr}\;\rho =1. \end{array}$$

Originally, ρ was called the density operator of the quantum state, but it has become customary for ρ also to be called a quantum state.

### 2.2. Photon Number State

The most typical quantum state of light is the photon number state |n〉, which represents a state in which the number of photons is *n*, and it forms the following orthonormal basis:

(2) $$\begin{array}{c} \sum_{n=0}^{\infty }|n\rangle \langle n|=I,\langle n|m\rangle =\delta_{mn}, \end{array}$$

where I is the identity operator on Hilbert space, and $\delta_{mn}$ is the Kronecker delta.

### 2.3. Coherent State

This paper considers a quasi-Bell state that has been constructed using coherent states that are nonorthogonal quantum states. The coherent state is known as the most fundamental quantum state of light, and this state is very important because it can be realized approximately using laser light. The coherent state is expressed as

(3) $$\begin{array}{c} |\alpha \rangle =\sum_{n=0}^{\infty }\frac{\alpha^{n}}{\sqrt{n!}}e^{-\frac{1}{2}{\left|\alpha \right|}^{2}}|n\rangle , \end{array}$$

where α is the complex amplitude of the coherent state, and the average number of photons of the state is ${\langle n\rangle }_{\mathrm{Coh}}={\left|\alpha \right|}^{2}$.

The inner product of the two coherent states corresponding to the amplitudes α and β is

(4) $$\begin{array}{c} \langle \alpha |\beta \rangle =e^{\alpha^{*}\beta -\frac{{\left|\alpha \right|}^{2}}{2}-\frac{{\left|\beta \right|}^{2}}{2}}. \end{array}$$

If both α and β are real numbers, then the value of 〈α|β〉 is also a real number. In addition, the coherent state that is reflected from a beam splitter with reflectivity *R* is subjected to energy attenuation, causing it to become another coherent state, represented by $|\sqrt{R}\alpha \rangle$.

### 2.4. Quasi-Bell State

The quasi-Bell state [12] is an entangled state that is constructed using nonorthogonal quantum states but has maximum entanglement. The two modes of the quasi-Bell state can be labeled S and A. In this paper, we use two coherent states as the nonorthogonal quantum states, where the coherent states with amplitudes α and β are denoted by |α〉 and |β〉. The quasi-Bell states are represented as the quantum states of the composite system SA as follows:

(5) $$\begin{array}{ccc} {|\Psi_{1}\rangle }_{\mathrm{SA}} & = & h_{1}\left({|\alpha \rangle }_{S}{|\beta \rangle }_{A}+{|-\alpha \rangle }_{S}{|-\beta \rangle }_{A}\right), \end{array}$$

(6) $$\begin{array}{ccc} {|\Psi_{2}\rangle }_{\mathrm{SA}} & = & h_{2}\left({|\alpha \rangle }_{S}{|\beta \rangle }_{A}-{|-\alpha \rangle }_{S}{|-\beta \rangle }_{A}\right), \end{array}$$

(7) $$\begin{array}{ccc} {|\Psi_{3}\rangle }_{\mathrm{SA}} & = & h_{3}\left({|\alpha \rangle }_{S}{|-\beta \rangle }_{A}+{|-\alpha \rangle }_{S}{|\beta \rangle }_{A}\right), \end{array}$$

(8) $$\begin{array}{ccc} {|\Psi_{4}\rangle }_{\mathrm{SA}} & = & h_{4}\left({|\alpha \rangle }_{S}{|-\beta \rangle }_{A}-{|-\alpha \rangle }_{S}{|\beta \rangle }_{A}\right), \end{array}$$

where

(9) $$\begin{array}{ccc} h_{1} & = & h_{3}=\frac{1}{\sqrt{2\left(1+\kappa_{S}\kappa_{A}\right)}}, \end{array}$$

(10) $$\begin{array}{ccc} h_{2} & = & h_{4}=\frac{1}{\sqrt{2\left(1-\kappa_{S}\kappa_{A}\right)}}, \end{array}$$

(11) $$\begin{array}{ccc} \kappa_{S} & = & \langle \alpha |-\alpha \rangle =\langle -\alpha |\alpha \rangle =e^{-{2|\alpha |}^{2}}, \end{array}$$

(12) $$\begin{array}{ccc} \kappa_{A} & = & \langle \beta |-\beta \rangle =\langle -\beta |\beta \rangle =e^{-{2|\beta |}^{2}}, \end{array}$$

and α and β are nonnegative real numbers.

${|\Psi_{2}\rangle }_{\mathrm{SA}}$ has the maximum entanglement, and the amount of entanglement is 1 ebit when α=β. Therefore, we treat ${|\Psi_{2}\rangle }_{\mathrm{SA}}$ with α=β as the maximum quasi-Bell state in this paper. The average number of photons in mode S is ${\langle n\rangle }_{\mathrm{Max}}={\left|\alpha \right|}^{2}{\coth(2|\alpha |}^{2})$, and the minimum average number of photons is 0.5 because ${\langle n\rangle }_{\mathrm{Max}}\to 0.5$ when α→0. Additionally, we treat ${|\Psi_{1}\rangle }_{\mathrm{SA}}$ with α=β as the non-maximum quasi-Bell state in this paper. Note that the amount of entanglement in this case is smaller than 1 ebit. The average number of photons in mode S is ${\langle n\rangle }_{\mathrm{NonMax}}={\left|\alpha \right|}^{2}{\tanh(2|\alpha |}^{2})$, and the minimum average number of photons is 0 because ${\langle n\rangle }_{\mathrm{NonMax}}\to 0$ when α→0.

### 2.5. TSVS

In reference [9], an *m*-fold tensor product of the following TSVS was used as an EPR state:

(13) $$\begin{array}{c} {|\psi \rangle }_{\mathrm{SA}}=\sum_{n=0}^{\infty }\sqrt{\frac{N_{S}^{n}}{{\left(N_{S}+1\right)}^{n+1}}}{|n\rangle }_{S}{|n\rangle }_{A}, \end{array}$$

where ${\langle n\rangle }_{\mathrm{TSVS}}=N_{S}$ represents the average number of photons in mode S. In this paper, we set m=1 and analyze the TSVS.

The TSVS is one of the most important entangled states and has been discussed in numerous studies as a basic quantum state in both quantum illumination [20] and quantum reading [6] protocols. In particular, the amount of entanglement of the TSVS, which is given by

(14) $$\begin{array}{c} E_{\mathrm{TSVS}}=\left(N_{S}+1\right)\log_{2}\left(N_{S}+1\right)-N_{S}\log_{2}N_{S}, \end{array}$$

can exceed 1 ebit. A larger average number of photons causes a greater amount of entanglement because $\lim_{N_{S}\to \infty }E_{\mathrm{TSVS}}=\infty$.

### 2.6. Asymmetric Classical Communication

In this paper, we also compare the proposed system with an ACC system that has no mode A—mode A can be considered to be the vacuum state ${|0\rangle }_{A}$ in an AQC system without entanglement. Here, the coherent state |α〉 is prepared by Bob as the light source and is directed toward one of the two beam splitters operated by Alice. By switching the two beam splitters with their reflectivities of $R_{0}$ and $R_{1}\left(R_{0}<R_{1}\right)$, Alice encodes binary information using the different reflectivities. The reflected light collected by Bob thus becomes the binary coherent state signal $\left\{|\sqrt{R_{0}}\alpha \rangle ,|\sqrt{R_{1}}\alpha \rangle \right\}$, and this can be considered to be an ASK modulation scheme. Assuming that the binary signal has equal *a priori* probabilities and that the measurement in Bob’s detector, which we call an optimum classical receiver, is a homodyne measurement, the error probability for Bob is then given as follows:

(15) $$\begin{array}{c} P_{e}^{\left(\mathrm{Hom}\right)}=\frac{1}{2}\left\{1+\mathrm{erf}\left(\frac{\sqrt{R_{0}{\left|\alpha \right|}^{2}}-\sqrt{R_{1}{\left|\alpha \right|}^{2}}}{\sqrt{2}}\right)\right\}. \end{array}$$

## 3. Model of an ASK-Type AQC System

In this section, we describe the model of the AQC system that is constructed using the entangled states and the description of Bob’s received quantum states in the AQC system.

**Figure 1:**
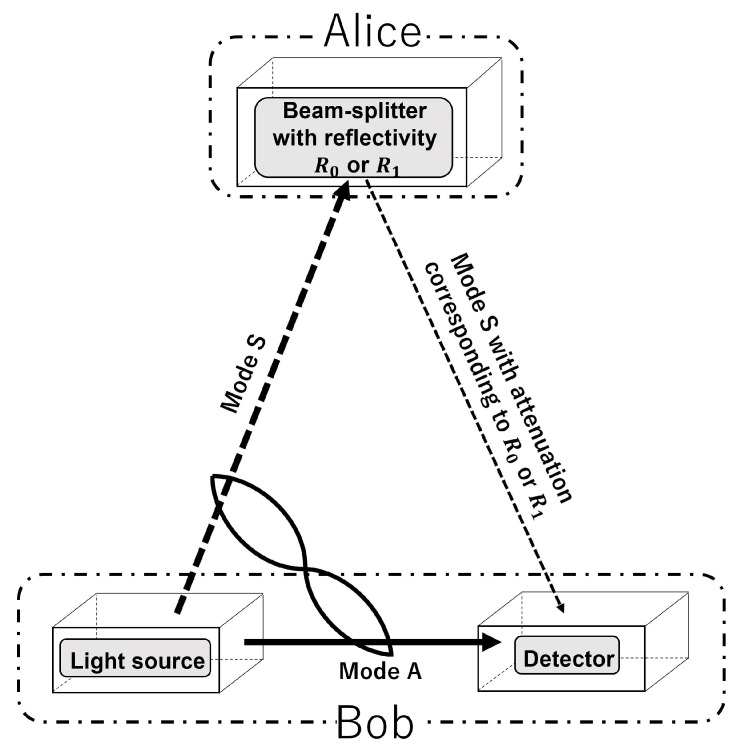
Protocol of an amplitude shift keying-type asymmetric quantum communication system.

A diagram of the model of the AQC system using the entangled state is shown in Figure 1. Binary information is sent from Alice to Bob. The dashed and solid arrows in the figure represent mode S and mode A of the entangled state, respectively. The AQC system protocol is given as follows:

**Protocol of an AQC system (Figure 1).**
  1. Bob (receiver) inputs the light corresponding to mode A directly into their detector.
  2. Bob radiates the light corresponding to mode S toward one of the two beam splitters operated by Alice (sender); Alice then switches the two beam splitters with reflectivities of $R_{0}$ and $R_{1}$$\left(R_{0}<R_{1}\right)$ to encode the binary information.
  3. The subsequently reflected light with reflectivity of either $R_{0}$ or $R_{1}$ is then collected by Bob’s detector.
  4. Bob decodes the binary information received at their detector by performing an optimum quantum measurement, i.e., a joint measurement of both light beams.

In this paper, we use the same model for a different purpose than that of reference [9], and therefore must first clarify the differences in this case when compared with the basic characteristics presented in [9]. We focus on investigation of Bob’s error performance as the basic characteristic of the ASK-type AQC system. In addition, the AQC system is characterized as an "ASK-type" system because Alice encodes information using different reflectivities, i.e., the differences in the amplitude of the reflected light corresponding to mode S.

### 3.1. Description of Received Quantum State

In this section, we describe the received quantum state corresponding to each entangled state. However, based on consideration of the prospect of the discussion, we analyze the quasi-Bell state using a Stinespring representation, and analyze the TSVS using a Kraus representation. Note that the light that corresponds to mode A is assumed to pass through an ideal channel because it is propagating inside Bob.

#### 3.1.1. Quasi-Bell State with Stinespring Representation

When a Stinespring representation is used, the loss incurred by an attenuated channel can be expressed using the interaction with the vacuum field as an environment mode E. In other words, the unitary evolution of the composite system SE can be described by applying the unitary operator $U_{\mathrm{SE}}^{\left(\eta \right)}$ to the SE, where the results represent the interaction between modes S and E:

(16) $$\begin{array}{ccc} U_{\mathrm{SE}}^{\left(\eta \right)}{|\alpha \rangle }_{S}{|0\rangle }_{E} & = & {|\sqrt{\eta }\alpha \rangle }_{S}{|\sqrt{1-\eta }\alpha \rangle }_{E}, \end{array}$$

(17) $$\begin{array}{ccc} U_{\mathrm{SE}}^{\left(\eta \right)}{|-\alpha \rangle }_{S}{|0\rangle }_{E} & = & {|-\sqrt{\eta }\alpha \rangle }_{S}{|-\sqrt{1-\eta }\alpha \rangle }_{E}, \end{array}$$

where η is the energy transmissivity. The reduced state of the composite system SAE on SA, i.e., the received quantum state, can be acquired by performing a partial trace over mode E, because only the composite system SA is actually measured by the detector.

For the case where b∈{0,1}, we now consider the received quantum states $\Psi_{\mathrm{SA}}^{\left(b\right)}$ when the binary information that was recorded using the reflectivities $R_{b}$ is denoted by “0” and “1”. Suppose that the transmitted quantum state at the light source is the maximum quasi-Bell state. Then, the received quantum state $\Psi_{\mathrm{SA}}^{\left(b\right)}$ can be represented by

(18) $$\begin{array}{c} \Psi_{\mathrm{SA}}^{\left(b\right)}=\;\mathrm{Tr}{\;}_{E}\left\{(U_{\mathrm{SE}}^{\left(R_{b}\right)}⊗I_{A}{|\Psi_{2}\rangle }_{\mathrm{SA}}\langle \Psi_{2}|⊗{|0\rangle }_{E}\langle 0|U_{\mathrm{SE}}^{\left(R_{b}\right)}⊗I_{A}{)}^{†}\right\}. \end{array}$$

The same supposition can be applied to the non-maximum quasi-Bell state.

#### 3.1.2. TSVS with Kraus Representation

In the TSVS case, a Kraus representation is used to express the attenuation channel. This Kraus representation allows us to describe the relationship between the transmitted and received quantum states, and can be expressed without use of an external system, unlike the Stinespring representation. If the transmitted and received quantum states are $\rho^{\left(\mathrm{in}\right)}$ and $\rho^{\left(\mathrm{out}\right)}$, respectively, then the Kraus representation of the attenuation channel is given as follows:

(19) $$\begin{array}{c} \rho^{\left(\mathrm{out}\right)}=\sum_{k=0}^{\infty }E_{k}^{\left(\eta \right)}\rho^{\left(\mathrm{in}\right)}{E_{k}^{\left(\eta \right)}}^{†}, \end{array}$$

where

(20) $$\begin{array}{c} E_{k}^{\left(\eta \right)}=\sum_{n}\sqrt{\left(\begin{array}{c} n\\ k \end{array}\right)}\sqrt{\eta^{n-k}{\left(1-\eta \right)}^{k}}|n-k\rangle \langle n| \end{array}$$

is the Kraus operator [21] for the attenuation channel with respect to the energy transmissivity η.

For the case where b∈{0,1}, we now consider the received quantum states $\psi_{\mathrm{SA}}^{\left(b\right)}$ when the binary information recorded using the reflectivities $R_{b}$ is denoted by “0” and “1”. The received quantum state $\psi_{\mathrm{SA}}^{\left(b\right)}$ can then be represented by

(21) $$\begin{array}{c} \psi_{\mathrm{SA}}^{\left(b\right)}=\sum_{k=0}^{\infty }\left\{{\left(E_{k}^{\left(R_{b}\right)}\right)}_{S}⊗I_{A}\right\}{|\psi \rangle }_{\mathrm{SA}}\langle \psi |{\left\{{\left(E_{k}^{\left(R_{b}\right)}\right)}_{S}⊗I_{A}\right\}}^{†}. \end{array}$$

### 3.2. Error Probability Determined by Optimum Quantum Measurement

The error probability is an important performance evaluation index for communication systems. In this paper, it is assumed that Bob has no information about the *a priori* probabilities $\xi_{b}\in \left\{\xi_{0},\xi_{1}\right\}$ that correspond to the binary information b∈{0,1}. If the *a priori* probabilities of the quantum states are unknown, it is known that use of the Bayes decision criterion with equal *a priori* probabilities under the quantum minimax criterion is the optimum approach from quantum detection theory [22]. If we suppose that the received quantum states are $\rho_{\mathrm{SA}}^{\left(0\right)}$ and $\rho_{\mathrm{SA}}^{\left(1\right)}$, and that the corresponding *a priori* probabilities are equal, i.e., $\xi_{0}=\xi_{1}=1/2$, then the error probability given by the optimum quantum measurement [23] is

(22) $$\begin{array}{c} P_{e}=\frac{1}{2}\left(1-\sum_{\lambda_{i}>0}\lambda_{i}\right), \end{array}$$

where $\left\{\lambda_{i}\right\}$ are the eigenvalues of $\rho_{\mathrm{SA}}^{\left(0\right)}-\rho_{\mathrm{SA}}^{\left(1\right)}$.

## 4. Derivation of Analytical Expression for the Error Probability of the Quasi-Bell State

In this section, we derive an analytical expression for the error probability of the AQC system when using the quasi-Bell state. As an example of the quasi-Bell state, we consider the case where the maximum quasi-Bell state is used. To calculate the error probability of given by Equation (22), it is necessary to obtain eigenvalues for the difference between the two received quantum states $\Psi_{\mathrm{SA}}^{\left(0\right)}-\Psi_{\mathrm{SA}}^{\left(1\right)}$. The coherent state is represented using either an infinite dimensional vector or an infinite matrix (i.e., the density operator). The maximum quasi-Bell state used here is constructed from the coherent states, and its density operator is thus infinite. The eigenvalues of $\Psi_{\mathrm{SA}}^{\left(0\right)}-\Psi_{\mathrm{SA}}^{\left(1\right)}$ also take the form of an infinite matrix and are generally difficult to calculate. However, in this paper, we find that the received quantum states $\Psi_{\mathrm{SA}}^{\left(0\right)}$ and $\Psi_{\mathrm{SA}}^{\left(1\right)}$ can be represented by an 8×8 matrix when a special orthonormal basis is used. Additionally, by deriving the eigenvalues for the 8×8 matrix of $\Psi_{\mathrm{SA}}^{\left(0\right)}-\Psi_{\mathrm{SA}}^{\left(1\right)}$, we can also derive an analytical expression for the error probability of the AQC system using the quasi-Bell state.

There are four steps that must be considered in the derivation of this analytical expression. Each step is explained individually below.

### 4.1. Step 1: Representation of the Received Quantum States by an 8 × 8 Matrix

First, if we focus on mode A in the received quantum states $\Psi_{\mathrm{SA}}^{\left(0\right)}$ and $\Psi_{\mathrm{SA}}^{\left(1\right)}$, we can see that the mode is constructed using the two coherent states $\left\{{|\pm \alpha \rangle }_{A}\right\}$. In the two-dimensional subspace of a Hilbert space that is spanned by {|±α〉}, the orthonormal basis $\left\{|\omega_{0}\rangle ,|\omega_{1}\rangle \right\}$ used in references [24,25] is the measurement state of the square-root measurement (SRM) [26], which is often applied to the representation of the numerical vector of the quasi-Bell state and to the representation of its density operator. It is known that this approach improves the prospects of the discussion (e.g., [27]). The SRM for $\left\{{|\pm \alpha \rangle }_{A}\right\}$ is an optimum quantum measurement that minimizes the error probability, and use of these measurement states means that $\left\{{|\pm \alpha \rangle }_{A}\right\}$ can be expressed as:

(23) $$\begin{array}{c} {|\pm \alpha \rangle }_{A}=\frac{1}{\varepsilon_{+}-\varepsilon_{-}}\left(\sqrt{\varepsilon_{\pm }}{|\omega_{0}\rangle }_{A}-\sqrt{\varepsilon_{\mp }}{|\omega_{1}\rangle }_{A}\right). \end{array}$$

Therefore, $\left\{{|\pm \alpha \rangle }_{A}\right\}$ can be represented by a two-dimensional vector, as follows:

(24) $$\begin{array}{c} {|\pm \alpha \rangle }_{A}=\frac{1}{\varepsilon_{+}-\varepsilon_{-}}\left[\begin{array}{c} \sqrt{\varepsilon_{\pm }}\\ -\sqrt{\varepsilon_{\mp }} \end{array}\right], \end{array}$$

where $\kappa_{A}=\kappa_{S}=:\kappa$ and $\varepsilon_{\pm }$ is given as follows:

(25) $$\begin{array}{c} \varepsilon_{\pm }=\frac{1\pm \sqrt{1-\kappa^{2}}}{2(1-\kappa^{2})}. \end{array}$$

Next, if we focus on mode A in the received quantum states $\Psi_{\mathrm{SA}}^{\left(0\right)}$ and $\Psi_{\mathrm{SA}}^{\left(1\right)}$, we can see that the mode is constructed using the following four coherent states: $\{{|-\sqrt{R_{1}}\alpha \rangle }_{S},{|-\sqrt{R_{0}}\alpha \rangle }_{S},{|\sqrt{R_{0}}\alpha \rangle }_{S},$${|\sqrt{R_{1}}\alpha \rangle }_{S}\}\left(=:\left\{|\alpha_{0}\rangle ,|\alpha_{1}\rangle ,|\alpha_{2}\rangle ,|\alpha_{3}\rangle \right\}\right)$. In the four-dimensional subspace of a Hilbert space that is spanned by these coherent states, we also consider the measurement state of the SRM to be an orthonormal basis. The representation of a four-dimensional vector of these coherent states in the four-dimensional subspace can be obtained immediately from the square root of their Gram matrix. In general, the Gram matrix for $\left\{|\alpha_{0}\rangle ,|\alpha_{1}\rangle ,|\alpha_{2}\rangle ,|\alpha_{3}\rangle \right\}$ is constructed as follows [28]:

(26) $$\begin{array}{c} \left[\begin{array}{cccc} \langle \alpha_{0}|\alpha_{0}\rangle & \langle \alpha_{0}|\alpha_{1}\rangle & \langle \alpha_{0}|\alpha_{2}\rangle & \langle \alpha_{0}|\alpha_{3}\rangle \\ \langle \alpha_{1}|\alpha_{0}\rangle & \langle \alpha_{1}|\alpha_{1}\rangle & \langle \alpha_{1}|\alpha_{2}\rangle & \langle \alpha_{1}|\alpha_{3}\rangle \\ \langle \alpha_{2}|\alpha_{0}\rangle & \langle \alpha_{2}|\alpha_{1}\rangle & \langle \alpha_{2}|\alpha_{2}\rangle & \langle \alpha_{2}|\alpha_{3}\rangle \\ \langle \alpha_{3}|\alpha_{0}\rangle & \langle \alpha_{3}|\alpha_{1}\rangle & \langle \alpha_{3}|\alpha_{2}\rangle & \langle \alpha_{3}|\alpha_{3}\rangle \end{array}\right]=\left[\begin{array}{cccc} 1 & b & c & d\\ b & 1 & a & c\\ c & a & 1 & b\\ d & c & b & 1 \end{array}\right], \end{array}$$

where

(27) $$\begin{array}{c} a=e^{-2R_{0}{\left|\alpha \right|}^{2}},\;\;\;b=e^{\sqrt{R_{0}R_{1}}{\left|\alpha \right|}^{2}-\frac{1}{2}{\left|\alpha \right|}^{2}\left(R_{0}+R_{1}\right)},\\ c=e^{-\sqrt{R_{0}R_{1}}{\left|\alpha \right|}^{2}-\frac{1}{2}{\left|\alpha \right|}^{2}\left(R_{0}+R_{1}\right)},\;\;\;d=e^{-2R_{1}{\left|\alpha \right|}^{2}}. \end{array}$$

However, because the representation of the square root of the Gram matrix is complicated, the method described in references [29,30,31] is applied in this paper. In references [29,30,31], the order of these coherent states was rearranged to be $\{{|-\sqrt{R_{0}}\alpha \rangle }_{S},{|\sqrt{R_{0}}\alpha \rangle }_{S},{|-\sqrt{R_{1}}\alpha \rangle }_{S},{|\sqrt{R_{1}}\alpha \rangle }_{S}\left(=:\left\{|\alpha_{0}^{\prime }\rangle ,|\alpha_{1}^{\prime }\rangle ,|\alpha_{2}^{\prime }\rangle ,|\alpha_{3}^{\prime }\rangle \right)\right\}$ to take advantage of the partial symmetry of the coherent state signal, and the Gram matrix Γ then became as follows:

(28) $$\begin{array}{c} \Gamma =\left[\begin{array}{cccc} \langle \alpha_{0}^{\prime }|\alpha_{0}^{\prime }\rangle & \langle \alpha_{0}^{\prime }|\alpha_{1}^{\prime }\rangle & \langle \alpha_{0}^{\prime }|\alpha_{2}^{\prime }\rangle & \langle \alpha_{0}^{\prime }|\alpha_{3}^{\prime }\rangle \\ \langle \alpha_{1}^{\prime }|\alpha_{0}^{\prime }\rangle & \langle \alpha_{1}^{\prime }|\alpha_{1}^{\prime }\rangle & \langle \alpha_{1}^{\prime }|\alpha_{2}^{\prime }\rangle & \langle \alpha_{1}^{\prime }|\alpha_{3}^{\prime }\rangle \\ \langle \alpha_{2}^{\prime }|\alpha_{0}^{\prime }\rangle & \langle \alpha_{2}^{\prime }|\alpha_{1}^{\prime }\rangle & \langle \alpha_{2}^{\prime }|\alpha_{2}^{\prime }\rangle & \langle \alpha_{2}^{\prime }|\alpha_{3}^{\prime }\rangle \\ \langle \alpha_{3}^{\prime }|\alpha_{0}^{\prime }\rangle & \langle \alpha_{3}^{\prime }|\alpha_{1}^{\prime }\rangle & \langle \alpha_{3}^{\prime }|\alpha_{2}^{\prime }\rangle & \langle \alpha_{3}^{\prime }|\alpha_{3}^{\prime }\rangle \end{array}\right]=\left[\begin{array}{cccc} 1 & a & b & c\\ a & 1 & c & b\\ b & c & 1 & d\\ c & b & d & 1 \end{array}\right]. \end{array}$$

Through observation of Γ, we found that it can be divided into four blocks using a 2×2 real symmetric matrix with the common structure of

(29) $$\begin{array}{c} M^{\left(2\right)}\left(u,v\right)=\left[\begin{array}{cc} u & v\\ v & u \end{array}\right]. \end{array}$$

The eigenvalues and corresponding orthonormal eigenvectors of $M^{\left(2\right)}\left(u,v\right)$ are given by

(30) $$\begin{array}{c} x_{1}=u+v,\;\;\;x_{2}=u-v \end{array}$$

and

(31) $$\begin{array}{c} |x_{1}\rangle =\frac{1}{\sqrt{2}}\left[\begin{array}{c} 1\\ 1 \end{array}\right],\;\;\;|x_{2}\rangle =\frac{1}{\sqrt{2}}\left[\begin{array}{c} 1\\ -1 \end{array}\right], \end{array}$$

respectively (Although $|x_{1}\rangle$ and $|x_{2}\rangle$ are not vectors in the Hilbert space of a quantum system, and in fact are numerical vectors, we use Dirac’s notation for convenience). We then obtain the spectral decomposition form of $M^{\left(2\right)}\left(u,v\right)$, which is expressed as

(32) $$\begin{array}{c} M^{\left(2\right)}\left(u,v\right)=\left(u+v\right)|x_{1}\rangle \langle x_{1}|+\left(u-v\right)|x_{2}\rangle \langle x_{2}|. \end{array}$$

Using $M^{\left(2\right)}\left(u,v\right)$, Γ can then be divided into blocks, as follows:

(33) $$\begin{array}{c} \Gamma =\left[\begin{array}{cc} M^{\left(2\right)}\left(1,a\right) & M^{\left(2\right)}\left(b,c\right)\\ M^{\left(2\right)}\left(b,c\right) & M^{\left(2\right)}\left(1,d\right) \end{array}\right]. \end{array}$$

By substituting the spectral decomposition form into the equation above, we obtain

(34) $$\begin{array}{c} \Gamma =M^{\left(+\right)}⊗|x_{1}\rangle \langle x_{1}|+M^{\left(-\right)}⊗|x_{2}\rangle \langle x_{2}|, \end{array}$$

where

(35) $$\begin{array}{c} M^{\left(+\right)}=\left[\begin{array}{cc} 1+a & b+c\\ b+c & 1+d \end{array}\right],\;\;\;M^{\left(-\right)}=\left[\begin{array}{cc} 1-a & b-c\\ b-c & 1-d \end{array}\right]. \end{array}$$

Both $M^{\left(+\right)}$ and $M^{\left(-\right)}$ have the common structure of

(36) $$\begin{array}{c} M^{\left(\pm \right)}=\left[\begin{array}{cc} p & q\\ q & r \end{array}\right], \end{array}$$

and the eigenvalues $\lambda_{\pm }\left(p,q,r\right)$ and the corresponding orthonormal eigenvectors $|\lambda_{\pm }\left(p,q,r\right)\rangle$ of this structure can be expressed as:

(37) $$\begin{array}{c} \lambda_{\pm }\left(p,q,r\right)=\frac{1}{2}\left(p+r\pm \sqrt{{\left(p-r\right)}^{2}+4q^{2}}\right) \end{array}$$

and

(38) $$\begin{array}{c} |\lambda_{\pm }\left(p,q,r\right)\rangle =\frac{|\lambda_{\pm }^{\prime }\left(p,q,r\right)\rangle }{\sqrt{\langle \lambda_{\pm }^{\prime }\left(p,q,r\right)|\lambda_{\pm }^{\prime }\left(p,q,r\right)\rangle }}, \end{array}$$

respectively, where

(39) $$\begin{array}{c} |\lambda_{\pm }^{\prime }\left(p,q,r\right)\rangle =\left[\begin{array}{c} p-r\pm \sqrt{{\left(p-r\right)}^{2}+4q^{2}}\\ 2q \end{array}\right]. \end{array}$$

Based on the derivation above, the analytical expressions for the eigenvalues $\left\{\lambda_{i}\right\}$ and the corresponding orthonormal eigenvectors $\left\{\lambda_{i}\right\}$ of Γ can be expressed as

(40) $$\begin{array}{cc} \lambda_{1} & =\lambda_{+}\left(1+a,b+c,1+d\right), \end{array}$$

(41) $$\begin{array}{cc} \lambda_{2} & =\lambda_{-}\left(1+a,b+c,1+d\right), \end{array}$$

(42) $$\begin{array}{cc} \lambda_{3} & =\lambda_{+}\left(1-a,b-c,1-d\right), \end{array}$$

(43) $$\begin{array}{cc} \lambda_{4} & =\lambda_{-}\left(1-a,b-c,1-d\right) \end{array}$$

and

(44) $$\begin{array}{cc} |\lambda_{1}\rangle & =|\lambda_{+}\left(1+a,b+c,1+d\right)\rangle ⊗|x_{1}\rangle , \end{array}$$

(45) $$\begin{array}{cc} |\lambda_{2}\rangle & =|\lambda_{-}\left(1+a,b+c,1+d\right)\rangle ⊗|x_{1}\rangle , \end{array}$$

(46) $$\begin{array}{cc} |\lambda_{3}\rangle & =|\lambda_{+}\left(1-a,b-c,1-d\right)\rangle ⊗|x_{2}\rangle , \end{array}$$

(47) $$\begin{array}{cc} |\lambda_{4}\rangle & =|\lambda_{-}\left(1-a,b-c,1-d\right)\rangle ⊗|x_{2}\rangle , \end{array}$$

respectively. Therefore, the spectral decomposition form of Γ is given as follows:

(48) $$\begin{array}{c} \Gamma =\sum_{i=1}^{4}\lambda_{i}|\lambda_{i}\rangle \langle \lambda_{i}|. \end{array}$$

Here, $\left\{|\lambda_{i}\rangle \right\}$ forms an orthonormal basis, which means that the square root of the Gram matrix, $\Gamma^{\frac{1}{2}}$, can be derived immediately as:

(49) $$\begin{array}{c} \Gamma^{\frac{1}{2}}=\sum_{i=1}^{4}\sqrt{\lambda_{i}}|\lambda_{i}\rangle \langle \lambda_{i}|=\left[\begin{array}{cccc} \gamma_{11}^{\left(+\right)} & \gamma_{11}^{\left(-\right)} & \gamma_{13}^{\left(+\right)} & \gamma_{13}^{\left(-\right)}\\ \gamma_{11}^{\left(-\right)} & \gamma_{11}^{\left(+\right)} & \gamma_{13}^{\left(-\right)} & \gamma_{13}^{\left(+\right)}\\ \gamma_{13}^{\left(+\right)} & \gamma_{13}^{\left(-\right)} & \gamma_{33}^{\left(+\right)} & \gamma_{33}^{\left(-\right)}\\ \gamma_{13}^{\left(-\right)} & \gamma_{13}^{\left(+\right)} & \gamma_{33}^{\left(-\right)} & \gamma_{33}^{\left(+\right)} \end{array}\right], \end{array}$$

where each element of the 4×4 matrix $\Gamma^{\frac{1}{2}}$ can be obtained directly via substitution of Equations (40)–(44) into $\sum_{i=1}^{4}\sqrt{\lambda_{i}}|\lambda_{i}\rangle \langle \lambda_{i}|$. See Appendix A for details. The four-dimensional vector representation of $\left\{{|-\sqrt{R_{0}}\alpha \rangle }_{S},{|\sqrt{R_{0}}\alpha \rangle }_{S},{|-\sqrt{R_{1}}\alpha \rangle }_{S},{|\sqrt{R_{1}}\alpha \rangle }_{S}\right\}$ is given as follows:

(50) $$\begin{array}{cc} \; & {|-\sqrt{R_{0}}\alpha \rangle }_{S}=\left[\begin{array}{c} \gamma_{11}^{\left(+\right)}\\ \gamma_{11}^{\left(-\right)}\\ \gamma_{13}^{\left(+\right)}\\ \gamma_{13}^{\left(-\right)} \end{array}\right],\;\;{|\sqrt{R_{0}}\alpha \rangle }_{S}=\left[\begin{array}{c} \gamma_{11}^{\left(-\right)}\\ \gamma_{11}^{\left(+\right)}\\ \gamma_{13}^{\left(-\right)}\\ \gamma_{13}^{\left(+\right)} \end{array}\right],\\ \; & {|-\sqrt{R_{1}}\alpha \rangle }_{S}=\left[\begin{array}{c} \gamma_{13}^{\left(+\right)}\\ \gamma_{13}^{\left(-\right)}\\ \gamma_{33}^{\left(+\right)}\\ \gamma_{33}^{\left(-\right)} \end{array}\right],\;\;{|\sqrt{R_{1}}\alpha \rangle }_{S}=\left[\begin{array}{c} \gamma_{13}^{\left(-\right)}\\ \gamma_{13}^{\left(+\right)}\\ \gamma_{33}^{\left(-\right)}\\ \gamma_{33}^{\left(+\right)} \end{array}\right]. \end{array}$$

Finally, by substituting Equations (24) and (50) into Equation (18), we obtain

(51) $$\begin{array}{ccc} \;\;\;\;\Psi_{\mathrm{SA}}^{\left(b\right)}\;\;\;\;\; & = & \;\;\;\;\;\;\mathrm{Tr}{\;}_{E}\left\{\left(U_{\mathrm{SE}}^{\left(R_{b}\right)}\;⊗\;I_{A}\right)\left({|\Psi_{2}\rangle }_{\mathrm{SA}}\langle \Psi_{2}|\;⊗\;{|0\rangle }_{E}\langle 0|\right){\left(U_{\mathrm{SE}}^{\left(R_{b}\right)}\;⊗\;I_{A}\right)}^{†}\right\}\\ \;\;\;\;\; & = & \;\;\;\;\;h_{2}^{2}\left({|\sqrt{R_{b}}\alpha \rangle }_{S}\langle \sqrt{R_{b}}\alpha |\;⊗\;{|\alpha \rangle }_{A}\langle \alpha |-L_{b}{|\sqrt{R_{b}}\alpha \rangle }_{S}\langle -\sqrt{R_{b}}\alpha |\;⊗\;{|\alpha \rangle }_{A}\langle -\alpha |\right.\\ & & \;\;\left.-L_{b}{|-\sqrt{R_{b}}\alpha \rangle }_{S}\langle \sqrt{R_{b}}\alpha |\;⊗\;{|-\alpha \rangle }_{A}\langle \alpha |+{|-\sqrt{R_{b}}\alpha \rangle }_{S}\langle -\sqrt{R_{b}}\alpha |\;⊗\;{|-\alpha \rangle }_{A}\langle -\alpha |\right), \end{array}$$

where $L_{b}=e^{-2\left(1-R_{b}\right){\left|\alpha \right|}^{2}}$, and by taking the difference between the two received quantum states, $\Psi_{\mathrm{SA}}^{\left(0\right)}-\Psi_{\mathrm{SA}}^{\left(1\right)}\left(=:U\right)$, we then obtain an 8×8 matrix that can be expressed as:

(52) $$\begin{array}{c} U=\Psi_{\mathrm{SA}}^{\left(0\right)}-\Psi_{\mathrm{SA}}^{\left(1\right)}=\left[\begin{array}{cccccccc} A & B & C & D & G & H & I & J\\ B & E & F & C & K & L & M & N\\ C & F & E & B & N & M & L & K\\ D & C & B & A & J & I & H & G\\ G & K & N & J & O & P & Q & R\\ H & L & M & I & P & S & T & Q\\ I & M & L & H & Q & T & S & P\\ J & N & K & G & R & Q & P & O \end{array}\right]. \end{array}$$

The 8×8 matrix *U* is a real symmetric matrix, and each element of this matrix can be obtained directly by substituting Equations (24) and (50) into Equation (52) and then taking the difference between $\Psi_{\mathrm{SA}}^{\left(0\right)}$ and $\Psi_{\mathrm{SA}}^{\left(1\right)}$. See Appendix B for details of this procedure.

### 4.2. Step 2: Similar Transformation of 8 × 8 Matrix

Next, we consider the derivation of the eigenvalues of the 8×8 real symmetric matrix *U*. In this paper, we derive the eigenvalues for the 8×8 real symmetry matrix *U* by using its symmetrical structure, although there is no general solution for the eigenvalues of a square matrix with an order of five or more because of the Abel–Ruffini theorem. To use the symmetrical structure of *U*, we must first convert *U* into a more tractable form.

When the eigenvalues of *U* and $U^{\prime }$ become equal when *U* is converted into $V^{-1}UV:=U^{\prime }$ using a regular matrix *V*; this is known as similarity transformation. Here, if such a similarity transformation of *U* is performed using the regular matrix

(53) $$\begin{array}{c} V=V^{-1}=\left[\begin{array}{cccccccc} 1 & 0 & 0 & 0 & 0 & 0 & 0 & 0\\ 0 & 0 & 0 & 1 & 0 & 0 & 0 & 0\\ 0 & 0 & 1 & 0 & 0 & 0 & 0 & 0\\ 0 & 1 & 0 & 0 & 0 & 0 & 0 & 0\\ 0 & 0 & 0 & 0 & 0 & 0 & 1 & 0\\ 0 & 0 & 0 & 0 & 0 & 1 & 0 & 0\\ 0 & 0 & 0 & 0 & 1 & 0 & 0 & 0\\ 0 & 0 & 0 & 0 & 0 & 0 & 0 & 1 \end{array}\right], \end{array}$$

we then obtain the matrix $U^{\prime }$ after the similarity transformation as follows:

(54) $$\begin{array}{c} U^{\prime }=V^{-1}UV=\left[\begin{array}{cccccccc} A & D & C & B & I & H & G & J\\ D & A & B & C & H & I & J & G\\ C & B & E & F & L & M & N & K\\ B & C & F & E & M & L & K & N\\ I & H & L & M & S & T & Q & P\\ H & I & M & L & T & S & P & Q\\ G & J & N & K & Q & P & O & R\\ J & G & K & N & P & Q & R & O \end{array}\right]. \end{array}$$

### 4.3. Step 3: Spectral Decomposition Form of the 8 × 8 Matrix

Through observation of $U^{\prime }$, we see that this matrix can be divided into 16 blocks using the 2×2 real symmetric matrix (29). We perform a spectral decomposition operation on $U^{\prime }$ in the same manner as Equation (34), and we then obtain

(55) $$\begin{array}{c} U^{\prime }=U^{\left(+\right)}⊗|x_{1}\rangle \langle x_{1}|+U^{\left(-\right)}⊗|x_{2}\rangle \langle x_{2}|, \end{array}$$

where

(56) $$\begin{array}{cc} U^{\left(+\right)} & =\left[\begin{array}{cccc} A+D & C+B & I+H & G+J\\ C+B & E+F & L+M & N+K\\ I+H & L+M & S+T & Q+P\\ G+J & N+K & Q+P & O+R \end{array}\right],\\ U^{\left(-\right)} & =\left[\begin{array}{cccc} A-D & C-B & I-H & G-J\\ C-B & E-F & L-M & N-K\\ I-H & L-M & S-T & Q-P\\ G-J & N-K & Q-P & O-R \end{array}\right] \end{array}$$

are also real symmetric matrices. Let the eigenvalues and eigenvectors of $U^{\left(+\right)}$ and $U^{\left(-\right)}$ be written as $\left\{\tau_{i}^{\left(+\right)}\right\}$, $\left\{\tau_{i}^{\left(-\right)}\right\}$ and $\left\{|\tau_{i}^{\left(+\right)}\rangle \right\}$, $\left\{|\tau_{i}^{\left(-\right)}\rangle \right\}$, respectively; then, we obtain:

(57) $$\begin{array}{c} U^{\left(+\right)}=\sum_{i=1}^{4}\tau_{i}^{\left(+\right)}|\tau_{i}^{\left(+\right)}\rangle \langle \tau_{i}^{\left(+\right)}|,\;\;\;U^{\left(-\right)}=\sum_{i=1}^{4}\tau_{i}^{\left(-\right)}|\tau_{i}^{\left(-\right)}\rangle \langle \tau_{i}^{\left(-\right)}|, \end{array}$$

because any real symmetric matrix can be spectrally decomposed. Substitution of these expressions into $U^{\prime }$ allows the spectral decomposition form of $U^{\prime }$ to be expressed as

(58) $$\begin{array}{c} U^{\prime }=\sum_{i=1}^{4}\tau_{i}^{\left(+\right)}|\tau_{i}^{\left(+\right)}\rangle \langle \tau_{i}^{\left(+\right)}|⊗|x_{1}\rangle \langle x_{1}|+\sum_{i=1}^{4}\tau_{i}^{\left(-\right)}|\tau_{i}^{\left(-\right)}\rangle \langle \tau_{i}^{\left(-\right)}|⊗|x_{2}\rangle \langle x_{2}|, \end{array}$$

where the eigenvalues of $U^{\prime }$ are given as follows:

(59) $$\begin{array}{c} \tau_{i}^{\left(+\right)},\;\;\;\tau_{i}^{\left(-\right)},\;\;\;i=\left\{1,2,3,4\right\}. \end{array}$$

### 4.4. Step 4: Eigenvalues of 4 × 4 Matrix

To determine the eigenvalues given by Equation (59), we must find the eigenvalues $\left\{\tau_{i}^{\left(+\right)}\right\}$ and $\left\{\tau_{i}^{\left(-\right)}\right\}$ of the 4×4 real symmetric matrices $U^{\left(+\right)}$ and $U^{\left(-\right)}$. All these eigenvalues must be real numbers because $U^{\left(+\right)}$ and $U^{\left(-\right)}$ are real symmetric matrices. The eigenvalues for $U^{\left(+\right)}$ and $U^{\left(-\right)}$ can be derived as follows.

The eigenvalues $\left\{\tau_{i}^{\left(+\right)}\right\}$ for the 4×4 real symmetric matrix $U^{\left(+\right)}$ are the solutions to the eigenvalue equation $\det\;(U^{\left(+\right)}-\tau_{i}^{\left(+\right)}I)=0$, where I is the identity matrix. We have

(60) $$\begin{array}{c} \det\;\left(U^{\left(+\right)}-\tau_{i}^{\left(+\right)}I\right)={\tau_{i}^{\left(+\right)}}^{2}\left({\tau_{i}^{\left(+\right)}}^{2}+A\tau_{i}^{\left(+\right)}+B\right)=0, \end{array}$$

where $U_{ij}^{\left(+\right)}$ is element (i,j) of $U^{\left(+\right)}$, and

(61) $$\begin{array}{cc} A= & -U_{11}^{\left(+\right)}-U_{22}^{\left(+\right)}-U_{33}^{\left(+\right)}-U_{44}^{\left(+\right)}\\ = & -\frac{\left(L_{0}+1\right)\left(a\kappa -1\right)+\left(1+L_{1}\right)\left(1-d\kappa \right)}{2(\kappa^{2}-1)},\\ B= & -{U_{12}^{\left(+\right)}}^{2}-{U_{13}^{\left(+\right)}}^{2}-{U_{14}^{\left(+\right)}}^{2}+U_{11}^{\left(+\right)}U_{22}^{\left(+\right)}-{U_{23}^{\left(+\right)}}^{2}-{U_{24}^{\left(+\right)}}^{2}+U_{11}^{\left(+\right)}U_{33}^{\left(+\right)}\\ \; & \;\;+U_{22}^{\left(+\right)}U_{33}^{\left(+\right)}-{U_{34}^{\left(+\right)}}^{2}+U_{11}^{\left(+\right)}U_{44}^{\left(+\right)}+U_{22}^{\left(+\right)}U_{44}^{\left(+\right)}+U_{33}^{\left(+\right)}U_{44}^{\left(+\right)} \end{array}$$

(62) $$\begin{array}{cc} = & -\frac{\left(L_{0}+1\right)\left(L_{1}+1\right)\left\{\left(a\kappa -1\right)\left(d\kappa -1\right)-{\left(b-c\kappa \right)}^{2}\right\}}{4{\left(\kappa^{2}-1\right)}^{2}}. \end{array}$$

We confirm directly that two of the eigenvalues $\left\{\tau_{i}^{\left(+\right)}\right\}$ are 0, and let $\tau_{3}^{\left(+\right)}=\tau_{4}^{\left(+\right)}=0$. To determine the signs of the other eigenvalues $\tau_{1}^{\left(+\right)}$ and $\tau_{2}^{\left(+\right)}$ for ${\tau_{i}^{\left(+\right)}}^{2}+A\tau_{i}^{\left(+\right)}+B=0$, we prove that $\tau_{1}^{\left(+\right)}\tau_{2}^{\left(+\right)}=B<0$ (see Appendix C for this proof), and we then know that these two eigenvalues have opposite signs: $\tau_{1}^{\left(+\right)}=\frac{-A+\sqrt{A^{2}-4B}}{2}>0$, and $\tau_{2}^{\left(+\right)}=\frac{-A-\sqrt{A^{2}-4B}}{2}<0$. Note that the corresponding eigenvalues $\left\{\tau_{i}^{\left(-\right)}\right\}$ for $U^{\left(-\right)}$ can be obtained in the same manner.

### 4.5. Derivation of Analytical Expression

The eigenvalues $\left\{\tau_{i}^{\left(+\right)}\right\}\cup \left\{\tau_{i}^{\left(-\right)}\right\}$ of $U^{\prime }$ (i.e., *U*) that were obtained from the analysis above can be substituted into Equation (22). Here, it can be confirmed that $\tau_{1}^{\left(+\right)},\tau_{1}^{\left(-\right)}>0$, and thus the error probability $P_{e}^{\left(\mathrm{Max}\right)}$ of the AQC system when using the maximum quasi-Bell state is finally given as follows:

(63) $$\begin{array}{cc} \;\;\;\;P_{e}^{\left(\mathrm{Max}\right)} & =\frac{1}{2}\left\{1-\frac{1}{2}\left(\sqrt{{\Lambda_{M}^{\left(+\right)}}^{2}-4\Xi_{M}^{\left(+\right)}}-\Lambda_{M}^{\left(+\right)}\right)-\frac{1}{2}\left(\sqrt{{\Lambda_{M}^{\left(-\right)}}^{2}-4\Xi_{M}^{\left(-\right)}}-\Lambda_{M}^{\left(-\right)}\right)\right\}, \end{array}$$

(64) $$\begin{array}{cc} \Lambda_{M}^{\left(\pm \right)} & =-\frac{\left(L_{0}\pm 1\right)\left(a\kappa \mp 1\right)+\left(1\pm L_{1}\right)\left(1\mp d\kappa \right)}{2(\kappa^{2}-1)}, \end{array}$$

(65) $$\begin{array}{cc} \Xi_{M}^{\left(\pm \right)} & =-\frac{\left(L_{0}\pm 1\right)\left(L_{1}\pm 1\right)\left\{\left(a\kappa \mp 1\right)\left(d\kappa \mp 1\right)-{\left(b\mp c\kappa \right)}^{2}\right\}}{4{\left(\kappa^{2}-1\right)}^{2}}. \end{array}$$

The error probability $P_{e}^{\left(\mathrm{NonMax}\right)}$ for the AQC system when using the non-maximum quasi-Bell state can also be obtained using steps 1 to 4 as per the case for the maximum quasi-Bell state. As a result, the corresponding probability is given by:

(66) $$\begin{array}{cc} \;\;\;\;P_{e}^{\left(\mathrm{NonMax}\right)} & =\frac{1}{2}\left\{1\;-\;\frac{1}{2}\left(\sqrt{{\Lambda_{N}^{\left(+\right)}}^{2}\;-\;4\Xi_{N}^{\left(+\right)}}\;-\;\Lambda_{N}^{\left(+\right)}\right)-\frac{1}{2}\left(\sqrt{{\Lambda_{N}^{\left(-\right)}}^{2}\;-\;4\Xi_{N}^{\left(-\right)}}\;-\;\Lambda_{N}^{\left(-\right)}\right)\right\}, \end{array}$$

(67) $$\begin{array}{cc} \Lambda_{N}^{\left(\pm \right)} & =-\frac{\left(L_{0}\mp 1\right)\left(a\kappa \mp 1\right)-\left(1\mp L_{1}\right)\left(1\mp d\kappa \right)}{2(\kappa^{2}+1)}, \end{array}$$

(68) $$\begin{array}{cc} \Xi_{N}^{\left(\pm \right)} & =-\frac{\left(L_{0}\mp 1\right)\left(L_{1}\mp 1\right)\left\{\left(a\kappa \mp 1\right)\left(d\kappa \mp 1\right)-{\left(b\mp c\kappa \right)}^{2}\right\}}{4{\left(\kappa^{2}+1\right)}^{2}}, \end{array}$$

where

(69) $$\begin{array}{cc} a & =e^{-2R_{0}{\left|\alpha \right|}^{2}},\;\;\;b=e^{\sqrt{R_{0}R_{1}}{\left|\alpha \right|}^{2}-\frac{1}{2}{\left|\alpha \right|}^{2}\left(R_{0}+R_{1}\right)},\;\;\;c=e^{-\sqrt{R_{0}R_{1}}{\left|\alpha \right|}^{2}-\frac{1}{2}{\left|\alpha \right|}^{2}\left(R_{0}+R_{1}\right)},\\ d & =e^{-2R_{1}{\left|\alpha \right|}^{2}},\;\;\;\kappa =e^{-{2|\alpha |}^{2}},\;\;\;L_{0}=e^{-2\left(1-R_{0}\right){\left|\alpha \right|}^{2}},\;\;\;L_{1}=e^{-2\left(1-R_{1}\right){\left|\alpha \right|}^{2}}. \end{array}$$

## 5. Error Performance

In this section, we present the results that were obtained numerically when using the error probability (15) for the ACC system and the analytical expression for the error probabilities (63) and (66) for the AQC system when using the quasi-Bell states. When using the TSVS, the calculation of the error probability $P_{e}^{\left(\mathrm{TSVS}\right)}$ in the case in which the average number of photons is small (${\langle n\rangle }_{\mathrm{TSVS}}\leq 5$) is performed based on the conventional numerical calculation method, i.e., using an equation that approximates Equation (13) to a finite value of *n*. In other words, as described in references [32,33], the calculation should be performed after suitable truncation of the Hilbert space by taking both the average number of photons and the order of the error probability into consideration. However, in the case where the average number of photons is large (i.e., ${\langle n\rangle }_{\mathrm{TSVS}}>5$), it is difficult to treat the eigenvalue problem in Equation (22) numerically because the dimensions of the received quantum state (i.e., its density operator) are large. Therefore, the AQC system’s performance is evaluated using the upper bound $P_{\mathrm{UB}}^{\left(\mathrm{TSVS}\right)}$ and the lower bound $P_{\mathrm{LB}}^{\left(\mathrm{TSVS}\right)}$ on the error probability $P_{e}^{\left(\mathrm{TSVS}\right)}$ given in reference [9] when the TSVS is used, as follows:

(70) $$\begin{array}{cc} P_{\mathrm{LB}}^{\left(\mathrm{TSVS}\right)} & =\frac{1}{2}\left[1-\sqrt{1-{\left\{\left(1-\sqrt{R_{0}R_{1}}-\sqrt{\left(1-R_{0}\right)\left(1-R_{1}\right)}\right)N_{S}+1\right\}}^{-2}}\right], \end{array}$$

(71) $$\begin{array}{cc} P_{\mathrm{UB}}^{\left(\mathrm{TSVS}\right)} & =\left\{\begin{array}{cc} \frac{1}{2}{\left\{\left(1-\sqrt{R_{0}}\right)N_{S}+1\right\}}^{-2} & \left(R_{1}=1\right)\\ \frac{1}{2}{\left(\Sigma \sigma^{z}-\Theta \theta^{z}\right)}^{-1} & \left(R_{1}\neq 1\right) \end{array}\right., \end{array}$$

where

(72) $$\begin{array}{cc} \sigma & =\frac{\left(1-R_{0}\right)N_{S}+1}{\left(1-R_{1}\right)N_{S}+1}, \end{array}$$

(73) $$\begin{array}{cc} \theta & =\frac{1-R_{0}}{1-R_{1}}, \end{array}$$

(74) $$\begin{array}{cc} \Sigma & =\frac{{\left\{\left(1-\sqrt{R_{0}R_{1}}\right)N_{S}+1\right\}}^{2}}{\left(1-R_{0}\right)N_{S}+1}, \end{array}$$

(75) $$\begin{array}{cc} \Theta & =\left(1-R_{1}\right)N_{S}, \end{array}$$

(76) $$\begin{array}{cc} z & =\left\{\begin{array}{cc} 0 & \left({\left(\Sigma -\Theta \right)}^{-2}\left(\ln\theta^{\Theta }-\ln\sigma^{\Sigma }\right)\geq 0\right)\\ 1 & \left({\left(\Sigma -\Theta \right)}^{-2}\left(\ln\theta^{\Theta }-\ln\sigma^{\Sigma }\right)<0\right) \end{array}\right.. \end{array}$$

This paper also provides a comparison with the universal lower bound $P_{U-\mathrm{LB}}$ on error probability given in reference [9] when the use of any multimode pure input state is permitted, as follows:

(77) $$\begin{array}{c} P_{U-\mathrm{LB}}=\frac{1}{2}\left\{1-\sqrt{1-{\left(\sqrt{R_{0}R_{1}}+\sqrt{\left(1-R_{0}\right)\left(1-R_{1}\right)}\right)}^{2N_{S}^{\prime }}}\right\}, \end{array}$$

where ${\langle n\rangle }_{U-\mathrm{LB}}=N_{S}^{\prime }$ is the average number of photons in mode S.

### 5.1. Performance Comparison of Use of Maximum and Non-Maximum Quasi-Bell States

**Figure 2:**
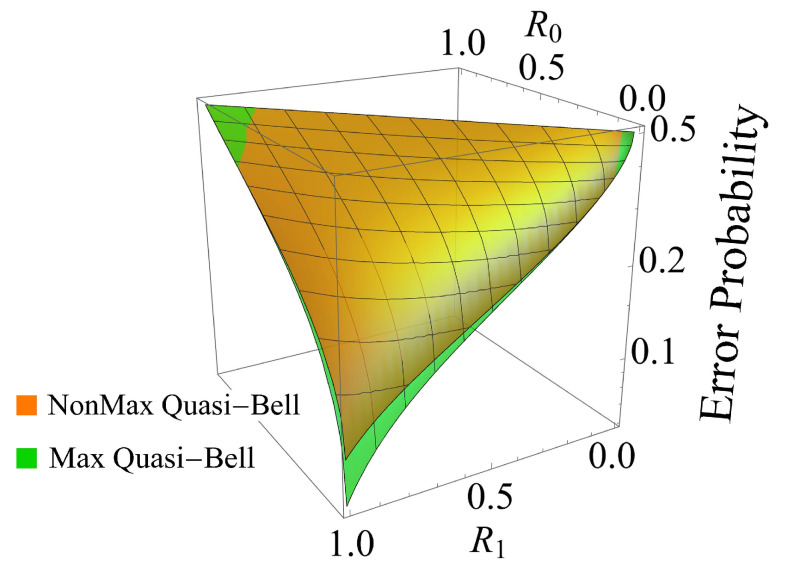
Error probability characteristics with respect to the reflectivities $\left\{R_{0},R_{1}\right\}$ when using the maximum quasi-Bell state (green curved surface) and the non-maximum quasi-Bell state (orange curved surface). The average number of photons is fixed at 1.

Figure 2 plots the error probabilities for the AQC system versus the reflectivities $R_{0}$ and $R_{1}$ ($R_{0}<R_{1}$), which are changed from 0 to 1 when the transmitted average number of photons in the maximum and non-maximum quasi-Bell states is fixed at ${\langle n\rangle }_{\mathrm{Max}}={\langle n\rangle }_{\mathrm{NonMax}}=1$. The error probability when the maximum quasi-Bell state is used is represented by the green curved surface, and the error probability when the non-maximum quasi-Bell state is used is represented by the orange curved surface.

**Figure 3:**
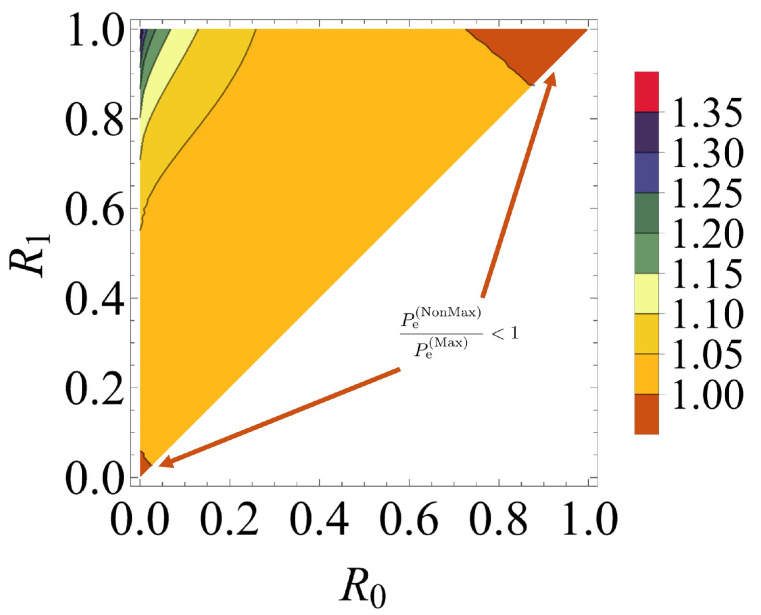
Contour plot of the ratio of the error probability when using the non-maximum quasi-Bell state ($P_{e}^{\left(\mathrm{NonMax}\right)}$) to that when using the maximum quasi-Bell state ($P_{e}^{\left(\mathrm{Max}\right)}$). The average number of photons is fixed at 1.

Figure 2 shows that the error probability $P_{e}^{\left(\mathrm{Max}\right)}$ when the maximum quasi-Bell state is used tends to decrease when $R_{1}-R_{0}$ increases. In particular, the figure shows reasonable results that confirm that the maximum error probability is achieved when $R_{0}\approx R_{1}$, and that the minimum error probability is achieved when $R_{0}=0$ and $R_{1}=1$. Comparison of the error probabilities of the AQC system when using the maximum and non-maximum quasi-Bell states shows that the maximum quasi-Bell state is superior in most cases, but there is no significant difference between the two cases. Furthermore, use of the maximum quasi-Bell state does not always provide superior results, because the AQC system using the non-maximum quasi-Bell state is superior to the corresponding system using the maximum quasi-Bell state in the extreme case in which $R_{1}-R_{0}$ is very small and $R_{0}\approx 1$ or $R_{0}\approx 0$. The details can be seen in Figure 3 regarding the contour plot of the ratio, $P_{e}^{\left(\mathrm{NonMax}\right)}/P_{e}^{\left(\mathrm{Max}\right)}$, of the error probability when using the non-maximum state to that when using the maximum quasi-Bell state. Figure 3 shows that the ratio is between 1 and 1.1 in most cases, but the ratio may be less than 1 in the extreme case.

Actually, there are several studies comparing the performance between the maximum and non-maximum quasi-Bell states, such as quantum teleportation [15], quantum superdense coding [34], quantum reading [33], and quantum illumination [18,19]. Reference [15] showed that the non-maximum quasi-Bell state offers an advantage over the maximum quasi-Bell state at small coherent amplitudes, and may offer more resistance to attenuation than the maximum quasi-Bell state. References [18,19,33,34] showed that the non-maximum quasi-Bell state offers more resistance to attenuation and phase noise than the maximum quasi-Bell state, in some special cases, and the result of this paper is one more piece of evidence in terms of that. That evidence also reveals a fact that the maximum quasi-Bell state offers better performance than the non-maximum quasi-Bell state in ideal cases, such as cases with large coherent amplitudes or environments without noise, but the opposite may be true in some unideal cases. Therefore, for some applications using the quasi-Bell state at small coherent amplitudes or environments with noise, the non-maximum quasi-Bell state which offers more resistance to noise may play an important role. We must be careful not to dismiss the value of using the non-maximum quasi-Bell state, although the reason in terms of its superiority has not yet been elucidated. We consider the issue to be an interesting topic that has value as a future subject.

### 5.2. Performance Comparison Using Each Quantum State

**Figure 4:**
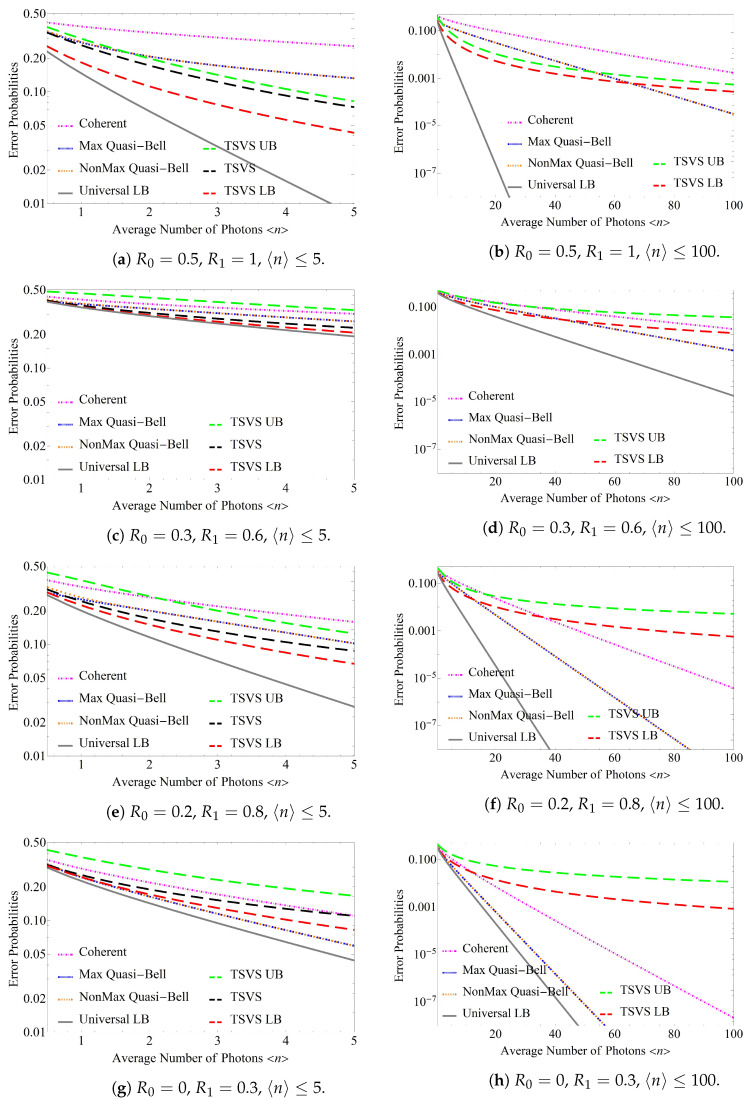
Error probabilities with respect to the average number of photons 〈n〉 when using the coherent state (denoted by Coherent), the maximum quasi-Bell state (denoted by Max Quasi-Bell), the non-maximum quasi-Bell state (denoted by NonMax Quasi-Bell), and the two-mode squeezed vacuum state (denoted by TSVS) with reflectivities $\left\{R_{0},R_{1}\right\}=\left\{0.5,1\right\},\left\{0.3,0.6\right\},\left\{0.2,0.8\right\},\left\{0,0.3\right\}$. 〈n〉 ranges up to either 5 or 100. Universal LB, TSVS UB, and TSVS LB present the universal lower bound on the error probability, the upper bound on the error probability for the TSVS case, and the lower bound on the error probability for the TSVS case, respectively.

Figure 4a,c,e,g shows the error probabilities obtained when the average number of photons $\langle n\rangle (:={\langle n\rangle }_{U-\mathrm{LB}}={\langle n\rangle }_{\mathrm{TSVS}}={\langle n\rangle }_{\mathrm{Max}}={\langle n\rangle }_{\mathrm{NonMax}}={\langle n\rangle }_{\mathrm{Coh}})$, which is regarded as the signal energy, is varied from 0.5 to 5 for the AQC system when using the TSVS, the maximum quasi-Bell state, and the non-maximum quasi-Bell state, and the ACC system using the coherent state, for which the reflectivities $\left\{R_{0},R_{1}\right\}$ were fixed at {0.5,1},{0.3,0.6},{0.2,0.8}, and {0,0.3}, respectively. The pink chain line represents the error probability $P_{e}^{\left(\mathrm{Hom}\right)}$ for the ACC system, and the blue and orange dotted lines represent the error probabilities $P_{e}^{\left(\mathrm{Max}\right)}$ and $P_{e}^{\left(\mathrm{NonMax}\right)}$ for the AQC system when using the maximum and non-maximum quasi-Bell states, respectively. The black dashed line represents the error probability $P_{e}^{\left(\mathrm{TSVS}\right)}$ for the AQC system when using the TSVS, and the green and red dashed lines represent the upper bound $P_{\mathrm{UB}}^{\left(\mathrm{TSVS}\right)}$ and the lower bound $P_{\mathrm{LB}}^{\left(\mathrm{TSVS}\right)}$, respectively, for $P_{e}^{\left(\mathrm{TSVS}\right)}$. The gray solid line represents the universal lower bound on the error probability, $P_{U-\mathrm{LB}}$. The horizontal axis represents the average number of photons, and the vertical axis represents the error probability in each case. The minimum average number of photons that can be considered is 0.5 because the minimum average number of photons in the maximum quasi-Bell state is ${\langle n\rangle }_{\mathrm{Max}}=0.5$, and cases smaller than that are not defined. If the average number of photons is greater than 5, it then becomes difficult to calculate the error probability $P_{e}^{\left(\mathrm{TSVS}\right)}$; therefore, only the upper bound $P_{\mathrm{UB}}^{\left(\mathrm{TSVS}\right)}$ and the lower bound $P_{\mathrm{LB}}^{\left(\mathrm{TSVS}\right)}$ are used for the evaluation of this probability. Figure 4b,d,f,h shows the error performances corresponding to those shown in Figure 4a,c,e,g, respectively, when the average number of photons 〈n〉 is increased from 0.5 to 100.

These figures confirm that the AQC system using the quasi-Bell state always maintains a clear performance advantage over the ACC system, despite increases in the average number of photons 〈n〉. Although the error probability of the AQC system when using the quasi-Bell state is similar to that of the ACC system, in that it decreases exponentially in tandem with the increase in 〈n〉, the AQC system can achieve the same error probability when using only half of the average number of photons used by the ACC system. Conversely, Figure 4b,d,f,h shows the error probability for the AQC system using the TSVS approaches that of the ACC system with increasing 〈n〉. In particular, as shown in Figure 4f, the ACC system provides superior performance to the AQC system using the TSVS when 〈n〉>35. Figure 4g,h shows that the performance of the ACC system exceeds that of the AQC system using the TSVS when the average number of photons is smaller, i.e., when 〈n〉≈5. This is contrary to the results presented in reference [9], which indicated that the performance obtained when using the *m*-shot EPR state exceeds that of the coherent state as the average number of photons increases, and this performance is considered to be a characteristic unique to the one-shot pulse case. Consideration of these figures together with Figure 4b,d shows that the ACC system exceeds the performance of the AQC system using the TSVS with smaller average number of photons when $R_{0}$ is small or when $R_{1}-R_{0}$ is large. Otherwise, the latter system can maintain its performance superiority over the former within the range of the small average number of photons. In addition, as shown in Figure 4f, the AQC system using the quasi-Bell state demonstrates superior performance to the same system using the TSVS when 〈n〉>10. Figure 4g,h shows that the AQC system using the quasi-Bell state has an error probability that is the same as or lower than that of the system using the TSVS. Consideration of these figures together with Figure 4b,d shows that the performance of the AQC system using the quasi-Bell state exceeds that of the system using the TSVS with smaller average number of photons when $R_{0}$ is small or when $R_{1}-R_{0}$ is large. Otherwise, the former system requires a larger average number of photons to surpass the performance of the latter.

### 5.3. Performance Comparison with Universal Lower Bound

Finally, we consider a performance comparison with the universal lower bound on the error probability. For example, when the average number of photons is small, the AQC system using the TSVS almost reaches the universal lower bound, as illustrated in Figure 4c. However, as Figure 4d shows, the gap between the lower bound on the error probability for the TSVS and the universal lower bound increases when the average number of photons increases. This differs from the results reported in reference [9], which stated that when the *m*-shot EPR state is used, the universal lower bound is almost always reached, and this is considered to be a characteristic unique to the one-shot pulse case. The characteristics of Figure 4b,f,h become more outstanding and the gap between the lower bound on the error probability for the TSVS and the universal lower bound increases rapidly as the average number of photons increases. In addition, Figure 4b shows that gap between the error probability for the AQC system using the quasi-Bell state and the universal lower bound may also increase rapidly in the same manner as the TSVS. However, Figure 4f shows that the error probability of the AQC system using the quasi-Bell state differs from that of the system using the TSVS in that the gap with respect to the universal lower bound only increases slowly as the average number of photons increases. Furthermore, as shown in Figure 4h, the error probability for the AQC system using the quasi-Bell state almost reaches the universal lower bound even when the average number of photons increases in the case where both $R_{0}$ and $R_{1}$ are small.

**Figure 5:**
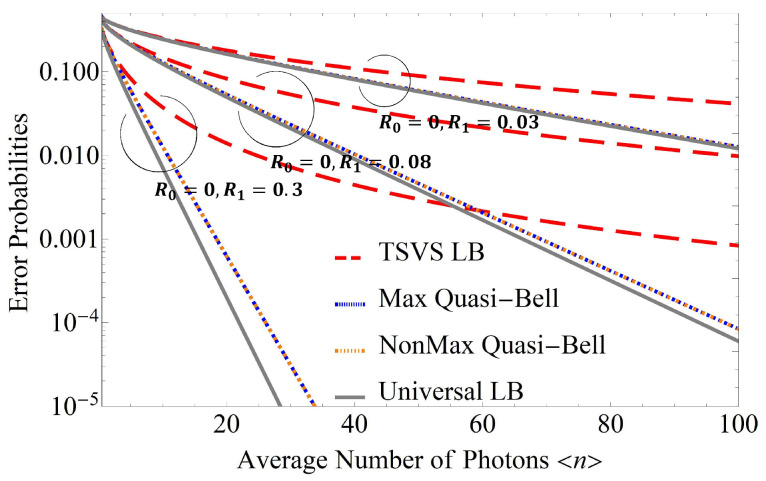
Error probabilities with respect to the average number of photons 〈n〉 when using the maximum quasi-Bell state (denoted by Max Quasi-Bell), the non-maximum quasi-Bell state (denoted by NonMax Quasi-Bell), and the two-mode squeezed vacuum state (denoted by TSVS) with reflectivities of $\left\{R_{0},R_{1}\right\}=\left\{0,0.3\right\},\left\{0,0.08\right\},\left\{0,0.03\right\}$. 〈n〉 ranges up to 100. Universal LB and TSVS LB represent the universal lower bound on the error probability and the lower bound on the error probability for TSVS, respectively.

**Figure 6:**
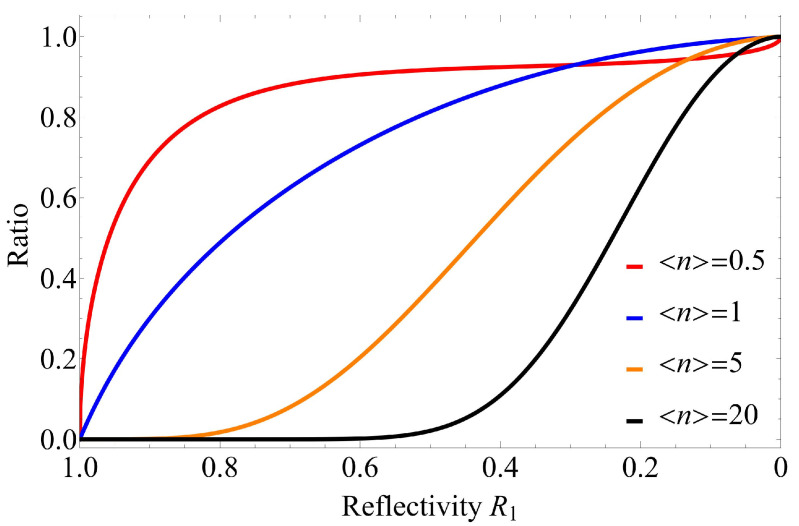
Ratio of the universal lower bound on the error probability to the error probability when using the maximum quasi-Bell state with respect to reflectivity $R_{1}$, where $R_{0}$ is fixed at 0.

To see these details of this characteristics in greater detail, Figure 5 shows the error performance when $\left\{R_{0},R_{1}\right\}=\left\{0,0.08\right\},\left\{0,0.03\right\}$ in addition to that when $\left\{R_{0},R_{1}\right\}=\left\{0,0.3\right\}$. As shown in Figure 5, $R_{0}$ is fixed at 0, and as $R_{1}$ decreases, the error probability for the AQC system using the quasi-Bell state becomes closer to the universal lower bound. To clearly demonstrate the trend of the gap between the error probability when using the quasi-Bell state and the universal lower bound, Figure 6 shows the ratio $P_{U-\mathrm{LB}}/P_{e}^{\left(\mathrm{Max}\right)}$ with respect to $R_{1}$ when fixing $R_{0}$ at 0. (Note the special case where the maximum quasi-Bell state becomes a Bell state when ${\langle n\rangle }_{\mathrm{Max}}=0.5$ [13].) As is evident in Figure 6, the ratio approaches 1 as $R_{1}$ decreases in spite of increasing 〈n〉. This performance characteristic makes it possible to use the quasi-Bell state to almost reach the universal lower bound in the AQC system, even if severe attenuation—where energy attenuation in the channel can be considered to be included in $R_{1}$—occurs in an ultra-long distance channel. (If energy attenuation associated with energy transmissivity η occurs in the channel, then just substitute $\eta R_{b}$ into $R_{b}$ in the results. For an example, Figure 4c expresses the error performance when $R_{0}=0.3$, $R_{1}=0.6$, and η=1; and when $R_{0}=0.5$, $R_{1}=0.75$, and η=0.8.)

## 6. Conclusions

In this paper, we have proposed an ASK-type AQC system as a step toward development of a new asymmetric communication system. In this AQC system, Bob, who has a high transmission capability, transmits one of the entangled light beams, which acts as a communication medium, to Alice, who has a low transmission capability; Alice operates on the light beam to encode the information that she wants to send to Bob, and then sends the light beam back to Bob. Bob then decodes the information received from Alice by performing an optimum quantum measurement (i.e., a joint measurement) of the other entangled light beam and the light beam that returned from Alice.

As a first step toward evaluation of the system performance, we focused on the communication performance from Alice to Bob, and investigated the basic performance characteristics based on the error probability criterion. First, using the quasi-Bell state as the light source, we derived an analytical expression for the error probability by using an 8×8 matrix representation to express the density operators of the two received quantum states affected by the reflectivities $\left\{R_{0},R_{1}\right\}$, which corresponded to the binary information that Alice wants to send. Then, using this analytical expression, we compared the superior performances of the AQC systems using the TSVS, the maximum quasi-Bell state, and the non-maximum quasi-Bell state with that of the asymmetric classical communication (ACC) system in terms of their error probabilities. As a result, it was clarified that the error probabilities of the AQC systems using the maximum and non-maximum quasi-Bell states differed only slightly. In addition, the error probability of the AQC system using the quasi-Bell state is always lower than that of the ACC system, regardless of the reflectivity setting, and the AQC system using the quasi-Bell state also shows a clear performance advantage over the system using the TSVS when a sufficiently large average number of photons is used. In fact, as described in Section 2, when the average number of photons is large, the amount of entanglement of the TSVS is overwhelmingly greater than that of the quasi-Bell state, but the results in this paper show that the performance of the AQC system using the quasi-Bell state is overwhelmingly better than that of the same system using the TSVS. Therefore, the performance of the AQC system should be determined by selecting a type of entangled state that is suitable for the system, rather than by considering the amount of entanglement. The conclusion above is strengthened by the fact that the performance of the ACC system surpassed that of the AQC system using the TSVS when the average number of photons was sufficiently large. What causes quasi-Bell state to work better than the TSVS? Unfortunately, as far as we know, there are no studies in terms of the comparison between the quasi-Bell state and the TSVS, except for references [18,19], although the reason for the superiority of the quasi-Bell state and some related potential properties of that have not been elucidated. These studies also reveal the fact that there are some entanglement-based systems or protocols that require a suitable entangled state rather than a large amount of entanglement to improve its performance. Additionally, we believe the advantage of the quasi-Bell state over the TSVS comes from the robustness of coherent states against attenuation. However, a perfect explanation is not yet available. We consider the issue to be a very challenging topic that has value as a future subject. Getting back to the main topic, when $R_{0}$ is small or when $R_{1}-R_{0}$ is large, the AQC system using the quasi-Bell states shows a clear advantage when only a small average number of photons is used. In particular, when $R_{0}=0$, the AQC system using the quasi-Bell state has the same or a lower error probability than the corresponding system using the TSVS. However, when $R_{1}=1$, a large average number of photons is required to enable the AQC system using the quasi-Bell state to exceed the performance of the system using the TSVS. Finally, we compared the error probability for the AQC system using the quasi-Bell state, the lower bound on the error probability for the AQC system using the TSVS, and the universal lower bound on the error probability, and found that performance closer to the universal lower bound was achieved using the quasi-Bell state when compared with the system with TSVS in the case when $R_{0}$ and $R_{1}$ are very small. As a result, it is expected that the AQC system using the quasi-Bell state will be applicable even in ultra-long distance channels, in which severe attenuation can occur.

In this paper, we evaluated the performance based on the error probability results for the AQC system and clarified the basic performance characteristics. In fact, if an eavesdropper Eve was present between Alice and Bob, one of the simplest attack methods for Eve would be to intercept the light corresponding to mode S, which is reflected from Alice. However, Eve cannot access the light corresponding to mode A, which remains inside Bob, and thus there would be a difference in reception performance between Eve and Bob. This reception performance difference creates the security of the AQC system. In addition, the AQC system discussed in this paper is expected to have various security applications, e.g., quantum cryptographic conferencing [35]. Future work will include security evaluation of this system, including the case of information leakage when an eavesdropper is present, and security enhancement for this system by performing some quantum communication protocols.

## Data Availability

Not applicable.

## Abbreviations

The following abbreviations are used in this manuscript:

AQC	Asymmetric quantum communication
ACC	Asymmetric classical communication
IoT	Internet of Things
EPR state	Einstein–Podolsky–Rosen state
TSVS	Two-mode squeezed vacuum state
ASK	Amplitude shift keying
Max Quasi-Bell	Maximum quasi-Bell state
NonMax Quasi-Bell	Non-maximum quasi-Bell state
Coherent	Coherent state
LB	Lower bound
UB	Upper bound
U-LB or Universal LB	Universal lower bound

## Appendix A

The elements $\left\{\gamma_{11}^{\left(\pm \right)},\gamma_{13}^{\left(\pm \right)},\gamma_{33}^{\left(\pm \right)}\right\}$ of $\Gamma^{\frac{1}{2}}$ are given as follows:

(A1) $$\begin{array}{cc} \gamma_{11}^{\left(\pm \right)}= & \sum_{i=0}^{1}\frac{\sqrt{{\left(-1\right)}^{i}e_{+}+a+d+2}{\left\{{\left(-1\right)}^{i}e_{+}+a-d\right\}}^{2}}{2\sqrt{2}\left[{\left\{{\left(-1\right)}^{i}e_{+}+a-d\right\}}^{2}+4{\left(b+c\right)}^{2}\right]}\\ \; & \pm \sum_{j=0}^{1}\frac{\sqrt{{\left(-1\right)}^{j}e_{-}-a-d+2}{\left\{{\left(-1\right)}^{j}e_{-}-a+d\right\}}^{2}}{2\sqrt{2}\left[{\left\{{\left(-1\right)}^{j}e_{-}-a+d\right\}}^{2}+4{\left(b-c\right)}^{2}\right]}, \end{array}$$

(A2) $$\begin{array}{cc} \gamma_{13}^{\left(\pm \right)}= & \sum_{i=0}^{1}\frac{\left(b+c\right)\sqrt{{\left(-1\right)}^{i}e_{+}+a+d+2}\left\{{\left(-1\right)}^{i}e_{+}+a-d\right\}}{\sqrt{2}\left[{\left\{{\left(-1\right)}^{i}e_{+}+a-d\right\}}^{2}+4{\left(b+c\right)}^{2}\right]}\\ \; & \pm \sum_{j=0}^{1}\frac{\left(b-c\right)\sqrt{{\left(-1\right)}^{j}e_{-}-a-d+2}\left\{{\left(-1\right)}^{j}e_{-}-a+d\right\}}{\sqrt{2}\left[{\left\{{\left(-1\right)}^{j}e_{-}-a+d\right\}}^{2}+4{\left(b-c\right)}^{2}\right]}, \end{array}$$

(A3) $$\begin{array}{cc} \gamma_{33}^{\left(\pm \right)}= & \sum_{i=0}^{1}\frac{\sqrt{2}{\left(b+c\right)}^{2}\sqrt{{\left(-1\right)}^{i}e_{+}+a+d+2}}{{\left\{{\left(-1\right)}^{i}e_{+}+a-d\right\}}^{2}+4{\left(b+c\right)}^{2}}\\ \; & \pm \sum_{j=0}^{1}\frac{\sqrt{2}{\left(b-c\right)}^{2}\sqrt{{\left(-1\right)}^{j}e_{-}-a-d+2}}{{\left\{{\left(-1\right)}^{j}e_{-}-a+d\right\}}^{2}+4{\left(b-c\right)}^{2}}, \end{array}$$

(A4) $$\begin{array}{cc} e_{\pm }= & \sqrt{{\left(\pm a\mp d\right)}^{2}+4{\left(b\pm c\right)}^{2}}. \end{array}$$

## Appendix B

The elements {A,…,T} of *U* are given as follows:

(A5) $$\begin{array}{cc} A= & \sqrt{\varepsilon_{-}}\sqrt{\varepsilon_{+}}\left(\gamma_{13}^{\left(-\right)}\gamma_{13}^{\left(+\right)}L_{1}-\gamma_{11}^{\left(-\right)}\gamma_{11}^{\left(+\right)}L_{0}\right)+\frac{1}{2}\varepsilon_{+}\left(\gamma_{11}^{\left(-\right)}-\gamma_{13}^{\left(-\right)}\right)\left(\gamma_{11}^{\left(-\right)}+\gamma_{13}^{\left(-\right)}\right)\\ \; & \;\;\;\;+\frac{1}{2}\varepsilon_{-}\left(\gamma_{11}^{\left(+\right)}-\gamma_{13}^{\left(+\right)}\right)\left(\gamma_{11}^{\left(+\right)}+\gamma_{13}^{\left(+\right)}\right), \end{array}$$

(A6) $$\begin{array}{cc} B= & \frac{1}{2}\left\{\sqrt{\varepsilon_{-}}\sqrt{\varepsilon_{+}}\left(-{\gamma_{11}^{\left(-\right)}}^{2}-{\gamma_{11}^{\left(+\right)}}^{2}+{\gamma_{13}^{\left(-\right)}}^{2}+{\gamma_{13}^{\left(+\right)}}^{2}\right)\right.\\ \; & \left.\;\;\;\;+\left(\varepsilon_{-}+\varepsilon_{+}\right)\left(\gamma_{11}^{\left(-\right)}\gamma_{11}^{\left(+\right)}L_{0}-\gamma_{13}^{\left(-\right)}\gamma_{13}^{\left(+\right)}L_{1}\right)\right\}, \end{array}$$

(A7) $$\begin{array}{cc} C= & \frac{1}{2}\left[\sqrt{\varepsilon_{-}}\sqrt{\varepsilon_{+}}\left\{L_{1}\left({\gamma_{13}^{\left(-\right)}}^{2}+{\gamma_{13}^{\left(+\right)}}^{2}\right)-L_{0}\left({\gamma_{11}^{\left(-\right)}}^{2}+{\gamma_{11}^{\left(+\right)}}^{2}\right)\right\}\right.\\ \; & \left.\;\;\;\;+\left(\varepsilon_{-}+\varepsilon_{+}\right)\left(\gamma_{11}^{\left(-\right)}\gamma_{11}^{\left(+\right)}-\gamma_{13}^{\left(-\right)}\gamma_{13}^{\left(+\right)}\right)\right], \end{array}$$

(A8) $$\begin{array}{cc} D= & \frac{1}{2}L_{0}\left({\gamma_{11}^{\left(-\right)}}^{2}\varepsilon_{+}+{\gamma_{11}^{\left(+\right)}}^{2}\varepsilon_{-}\right)+\sqrt{\varepsilon_{-}}\sqrt{\varepsilon_{+}}\left(\gamma_{13}^{\left(-\right)}\gamma_{13}^{\left(+\right)}-\gamma_{11}^{\left(-\right)}\gamma_{11}^{\left(+\right)}\right)\\ \; & \;\;\;\;-\frac{1}{2}L_{1}\left({\gamma_{13}^{\left(-\right)}}^{2}\varepsilon_{+}+{\gamma_{13}^{\left(+\right)}}^{2}\varepsilon_{-}\right), \end{array}$$

(A9) $$\begin{array}{cc} E= & \sqrt{\varepsilon_{-}}\sqrt{\varepsilon_{+}}\left(\gamma_{13}^{\left(-\right)}\gamma_{13}^{\left(+\right)}L_{1}-\gamma_{11}^{\left(-\right)}\gamma_{11}^{\left(+\right)}L_{0}\right)+\frac{1}{2}\varepsilon_{-}\left(\gamma_{11}^{\left(-\right)}-\gamma_{13}^{\left(-\right)}\right)\left(\gamma_{11}^{\left(-\right)}+\gamma_{13}^{\left(-\right)}\right)\\ \; & \;\;\;\;+\frac{1}{2}\varepsilon_{+}\left(\gamma_{11}^{\left(+\right)}-\gamma_{13}^{\left(+\right)}\right)\left(\gamma_{11}^{\left(+\right)}+\gamma_{13}^{\left(+\right)}\right), \end{array}$$

(A10) $$\begin{array}{cc} F= & \frac{1}{2}L_{0}\left({\gamma_{11}^{\left(-\right)}}^{2}\varepsilon_{-}+{\gamma_{11}^{\left(+\right)}}^{2}\varepsilon_{+}\right)+\sqrt{\varepsilon_{-}}\sqrt{\varepsilon_{+}}\left(\gamma_{13}^{\left(-\right)}\gamma_{13}^{\left(+\right)}-\gamma_{11}^{\left(-\right)}\gamma_{11}^{\left(+\right)}\right)\\ \; & \;\;\;\;-\frac{1}{2}L_{1}\left({\gamma_{13}^{\left(-\right)}}^{2}\varepsilon_{-}+{\gamma_{13}^{\left(+\right)}}^{2}\varepsilon_{+}\right), \end{array}$$

(A11) $$\begin{array}{cc} G= & \frac{1}{2}\left[\sqrt{\varepsilon_{-}}\sqrt{\varepsilon_{+}}\left\{L_{1}\left(\gamma_{13}^{\left(-\right)}\gamma_{33}^{\left(+\right)}+\gamma_{13}^{\left(+\right)}\gamma_{33}^{\left(-\right)}\right)-L_{0}\left(\gamma_{11}^{\left(-\right)}\gamma_{13}^{\left(+\right)}+\gamma_{11}^{\left(+\right)}\gamma_{13}^{\left(-\right)}\right)\right\}\right.\\ \; & \;\;\;\;\left.+\gamma_{13}^{\left(-\right)}\varepsilon_{+}\left(\gamma_{11}^{\left(-\right)}-\gamma_{33}^{\left(-\right)}\right)+\gamma_{13}^{\left(+\right)}\varepsilon_{-}\left(\gamma_{11}^{\left(+\right)}-\gamma_{33}^{\left(+\right)}\right)\right], \end{array}$$

(A12) $$\begin{array}{cc} H= & \frac{1}{2}\left\{\sqrt{\varepsilon_{-}}\sqrt{\varepsilon_{+}}\left(-\gamma_{11}^{\left(-\right)}\gamma_{13}^{\left(-\right)}-\gamma_{11}^{\left(+\right)}\gamma_{13}^{\left(+\right)}+\gamma_{13}^{\left(-\right)}\gamma_{33}^{\left(-\right)}+\gamma_{13}^{\left(+\right)}\gamma_{33}^{\left(+\right)}\right)\right.\\ \; & \;\;\;\;\left.+L_{0}\left(\gamma_{11}^{\left(-\right)}\gamma_{13}^{\left(+\right)}\varepsilon_{+}+\gamma_{11}^{\left(+\right)}\gamma_{13}^{\left(-\right)}\varepsilon_{-}\right)-L_{1}\left(\gamma_{13}^{\left(-\right)}\gamma_{33}^{\left(+\right)}\varepsilon_{+}+\gamma_{13}^{\left(+\right)}\gamma_{33}^{\left(-\right)}\varepsilon_{-}\right)\right\}, \end{array}$$

(A13) $$\begin{array}{cc} I= & \frac{1}{2}\left[\sqrt{\varepsilon_{-}}\sqrt{\varepsilon_{+}}\left\{L_{1}\left(\gamma_{13}^{\left(-\right)}\gamma_{33}^{\left(-\right)}+\gamma_{13}^{\left(+\right)}\gamma_{33}^{\left(+\right)}\right)-L_{0}\left(\gamma_{11}^{\left(-\right)}\gamma_{13}^{\left(-\right)}+\gamma_{11}^{\left(+\right)}\gamma_{13}^{\left(+\right)}\right)\right\}\right.\\ \; & \;\;\;\;\left.+\gamma_{11}^{\left(-\right)}\gamma_{13}^{\left(+\right)}\varepsilon_{+}+\gamma_{11}^{\left(+\right)}\gamma_{13}^{\left(-\right)}\varepsilon_{-}-\gamma_{13}^{\left(-\right)}\gamma_{33}^{\left(+\right)}\varepsilon_{+}-\gamma_{13}^{\left(+\right)}\gamma_{33}^{\left(-\right)}\varepsilon_{-}\right], \end{array}$$

(A14) $$\begin{array}{cc} J= & \frac{1}{2}\left\{\sqrt{\varepsilon_{-}}\sqrt{\varepsilon_{+}}\left(-\gamma_{11}^{\left(-\right)}\gamma_{13}^{\left(+\right)}-\gamma_{11}^{\left(+\right)}\gamma_{13}^{\left(-\right)}+\gamma_{13}^{\left(-\right)}\gamma_{33}^{\left(+\right)}+\gamma_{13}^{\left(+\right)}\gamma_{33}^{\left(-\right)}\right)\right.\\ \; & \;\;\;\;\left.+L_{0}\left(\gamma_{11}^{\left(-\right)}\gamma_{13}^{\left(-\right)}\varepsilon_{+}+\gamma_{11}^{\left(+\right)}\gamma_{13}^{\left(+\right)}\varepsilon_{-}\right)-L_{1}\left(\gamma_{13}^{\left(-\right)}\gamma_{33}^{\left(-\right)}\varepsilon_{+}+\gamma_{13}^{\left(+\right)}\gamma_{33}^{\left(+\right)}\varepsilon_{-}\right)\right\}, \end{array}$$

(A15) $$\begin{array}{cc} K= & \frac{1}{2}\left\{\sqrt{\varepsilon_{-}}\sqrt{\varepsilon_{+}}\left(-\gamma_{11}^{\left(-\right)}\gamma_{13}^{\left(-\right)}-\gamma_{11}^{\left(+\right)}\gamma_{13}^{\left(+\right)}+\gamma_{13}^{\left(-\right)}\gamma_{33}^{\left(-\right)}+\gamma_{13}^{\left(+\right)}\gamma_{33}^{\left(+\right)}\right)\right.\\ \; & \;\;\;\;\left.+L_{0}\left(\gamma_{11}^{\left(-\right)}\gamma_{13}^{\left(+\right)}\varepsilon_{-}+\gamma_{11}^{\left(+\right)}\gamma_{13}^{\left(-\right)}\varepsilon_{+}\right)-L_{1}\left(\gamma_{13}^{\left(-\right)}\gamma_{33}^{\left(+\right)}\varepsilon_{-}+\gamma_{13}^{\left(+\right)}\gamma_{33}^{\left(-\right)}\varepsilon_{+}\right)\right\}, \end{array}$$

(A16) $$\begin{array}{cc} L= & \frac{1}{2}\left[\sqrt{\varepsilon_{-}}\sqrt{\varepsilon_{+}}\left\{L_{1}\left(\gamma_{13}^{\left(-\right)}\gamma_{33}^{\left(+\right)}+\gamma_{13}^{\left(+\right)}\gamma_{33}^{\left(-\right)}\right)-L_{0}\left(\gamma_{11}^{\left(-\right)}\gamma_{13}^{\left(+\right)}+\gamma_{11}^{\left(+\right)}\gamma_{13}^{\left(-\right)}\right)\right\}\right.\\ \; & \;\;\;\;\left.+\gamma_{13}^{\left(-\right)}\varepsilon_{-}\left(\gamma_{11}^{\left(-\right)}-\gamma_{33}^{\left(-\right)}\right)+\gamma_{13}^{\left(+\right)}\varepsilon_{+}\left(\gamma_{11}^{\left(+\right)}-\gamma_{33}^{\left(+\right)}\right)\right], \end{array}$$

(A17) $$\begin{array}{cc} M= & \frac{1}{2}\left\{\sqrt{\varepsilon_{-}}\sqrt{\varepsilon_{+}}\left(-\gamma_{11}^{\left(-\right)}\gamma_{13}^{\left(+\right)}-\gamma_{11}^{\left(+\right)}\gamma_{13}^{\left(-\right)}+\gamma_{13}^{\left(-\right)}\gamma_{33}^{\left(+\right)}+\gamma_{13}^{\left(+\right)}\gamma_{33}^{\left(-\right)}\right)\right.\\ \; & \;\;\;\;\left.+L_{0}\left(\gamma_{11}^{\left(-\right)}\gamma_{13}^{\left(-\right)}\varepsilon_{-}+\gamma_{11}^{\left(+\right)}\gamma_{13}^{\left(+\right)}\varepsilon_{+}\right)-L_{1}\left(\gamma_{13}^{\left(-\right)}\gamma_{33}^{\left(-\right)}\varepsilon_{-}+\gamma_{13}^{\left(+\right)}\gamma_{33}^{\left(+\right)}\varepsilon_{+}\right)\right\}, \end{array}$$

(A18) $$\begin{array}{cc} N= & \frac{1}{2}\left[\sqrt{\varepsilon_{-}}\sqrt{\varepsilon_{+}}\left\{L_{1}\left(\gamma_{13}^{\left(-\right)}\gamma_{33}^{\left(-\right)}+\gamma_{13}^{\left(+\right)}\gamma_{33}^{\left(+\right)}\right)-L_{0}\left(\gamma_{11}^{\left(-\right)}\gamma_{13}^{\left(-\right)}+\gamma_{11}^{\left(+\right)}\gamma_{13}^{\left(+\right)}\right)\right\}\right.\\ \; & \;\;\;\;\left.+\gamma_{11}^{\left(-\right)}\gamma_{13}^{\left(+\right)}\varepsilon_{-}+\gamma_{11}^{\left(+\right)}\gamma_{13}^{\left(-\right)}\varepsilon_{+}-\gamma_{13}^{\left(-\right)}\gamma_{33}^{\left(+\right)}\varepsilon_{-}-\gamma_{13}^{\left(+\right)}\gamma_{33}^{\left(-\right)}\varepsilon_{+}\right], \end{array}$$

(A19) $$\begin{array}{cc} O= & \sqrt{\varepsilon_{-}}\sqrt{\varepsilon_{+}}\left(\gamma_{33}^{\left(-\right)}\gamma_{33}^{\left(+\right)}L_{1}-\gamma_{13}^{\left(-\right)}\gamma_{13}^{\left(+\right)}L_{0}\right)+\frac{1}{2}\varepsilon_{+}\left(\gamma_{13}^{\left(-\right)}-\gamma_{33}^{\left(-\right)}\right)\left(\gamma_{13}^{\left(-\right)}+\gamma_{33}^{\left(-\right)}\right)\\ \; & \;\;\;\;+\frac{1}{2}\varepsilon_{-}\left(\gamma_{13}^{\left(+\right)}-\gamma_{33}^{\left(+\right)}\right)\left(\gamma_{13}^{\left(+\right)}+\gamma_{33}^{\left(+\right)}\right), \end{array}$$

(A20) $$\begin{array}{cc} P= & \frac{1}{2}\left\{\sqrt{\varepsilon_{-}}\sqrt{\varepsilon_{+}}\left(-{\gamma_{13}^{\left(-\right)}}^{2}-{\gamma_{13}^{\left(+\right)}}^{2}+{\gamma_{33}^{\left(-\right)}}^{2}+{\gamma_{33}^{\left(+\right)}}^{2}\right)\right.\\ \; & \;\;\;\;\left.+\left(\varepsilon_{-}+\varepsilon_{+}\right)\left(\gamma_{13}^{\left(-\right)}\gamma_{13}^{\left(+\right)}L_{0}-\gamma_{33}^{\left(-\right)}\gamma_{33}^{\left(+\right)}L_{1}\right)\right\}, \end{array}$$

(A21) $$\begin{array}{cc} Q= & \frac{1}{2}\left[\sqrt{\varepsilon_{-}}\sqrt{\varepsilon_{+}}\left\{L_{1}\left({\gamma_{33}^{\left(-\right)}}^{2}+{\gamma_{33}^{\left(+\right)}}^{2}\right)-L_{0}\left({\gamma_{13}^{\left(-\right)}}^{2}+{\gamma_{13}^{\left(+\right)}}^{2}\right)\right\}\right.\\ \; & \;\;\;\;\left.+\left(\varepsilon_{-}+\varepsilon_{+}\right)\left(\gamma_{13}^{\left(-\right)}\gamma_{13}^{\left(+\right)}-\gamma_{33}^{\left(-\right)}\gamma_{33}^{\left(+\right)}\right)\right], \end{array}$$

(A22) $$\begin{array}{cc} R= & \frac{1}{2}L_{0}\left({\gamma_{13}^{\left(-\right)}}^{2}\varepsilon_{+}+{\gamma_{13}^{\left(+\right)}}^{2}\varepsilon_{-}\right)+\sqrt{\varepsilon_{-}}\sqrt{\varepsilon_{+}}\left(\gamma_{33}^{\left(-\right)}\gamma_{33}^{\left(+\right)}-\gamma_{13}^{\left(-\right)}\gamma_{13}^{\left(+\right)}\right)\\ \; & \;\;\;\;-\frac{1}{2}L_{1}\left({\gamma_{33}^{\left(-\right)}}^{2}\varepsilon_{+}+{\gamma_{33}^{\left(+\right)}}^{2}\varepsilon_{-}\right), \end{array}$$

(A23) $$\begin{array}{cc} S= & \sqrt{\varepsilon_{-}}\sqrt{\varepsilon_{+}}\left(\gamma_{33}^{\left(-\right)}\gamma_{33}^{\left(+\right)}L_{1}-\gamma_{13}^{\left(-\right)}\gamma_{13}^{\left(+\right)}L_{0}\right)+\frac{1}{2}\varepsilon_{-}\left(\gamma_{13}^{\left(-\right)}-\gamma_{33}^{\left(-\right)}\right)\left(\gamma_{13}^{\left(-\right)}+\gamma_{33}^{\left(-\right)}\right)\\ \; & \;\;\;\;+\frac{1}{2}\varepsilon_{+}\left(\gamma_{13}^{\left(+\right)}-\gamma_{33}^{\left(+\right)}\right)\left(\gamma_{13}^{\left(+\right)}+\gamma_{33}^{\left(+\right)}\right), \end{array}$$

(A24) $$\begin{array}{cc} T= & \frac{1}{2}L_{0}\left({\gamma_{13}^{\left(-\right)}}^{2}\varepsilon_{-}+{\gamma_{13}^{\left(+\right)}}^{2}\varepsilon_{+}\right)+\sqrt{\varepsilon_{-}}\sqrt{\varepsilon_{+}}\left(\gamma_{33}^{\left(-\right)}\gamma_{33}^{\left(+\right)}-\gamma_{13}^{\left(-\right)}\gamma_{13}^{\left(+\right)}\right)\\ \; & \;\;\;\;-\frac{1}{2}L_{1}\left({\gamma_{33}^{\left(-\right)}}^{2}\varepsilon_{-}+{\gamma_{33}^{\left(+\right)}}^{2}\varepsilon_{+}\right). \end{array}$$

## Appendix C

The proof that B<0 is presented as follows:

**Proof.** To prove that $B=-\frac{\left(L_{0}+1\right)\left(L_{1}+1\right)\left\{\left(a\kappa -1\right)\left(d\kappa -1\right)-{\left(b-c\kappa \right)}^{2}\right\}}{4{\left(\kappa^{2}-1\right)}^{2}}<0$, it is necessary to prove that

(A25) $$\begin{array}{c} \left(a\kappa -1\right)\left(d\kappa -1\right)-{\left(b-c\kappa \right)}^{2}>0, \end{array}$$

because $-\frac{\left(L_{0}+1\right)\left(L_{1}+1\right)}{4{\left(\kappa^{2}-1\right)}^{2}}<0$. This inequality can be rewritten as follows using a hyperbolic function:

(A26) $$\begin{array}{c} -2e^{-{\left|\alpha \right|}^{2}\left(2+R_{0}+R_{1}\right)}W>0, \end{array}$$

where

(A27) $$\begin{array}{c} W\;=\;\cosh\left\{{\left|\alpha \right|}^{2}\left(R_{0}\;-\;R_{1}\right)\right\}\;-\;\cosh\left\{{\left|\alpha \right|}^{2}\left(2\;+\;R_{0}\;+\;R_{1}\right)\right\}\;-\;1\;+\;\cosh\left\{{2|\alpha |}^{2}\left(1\;+\;\sqrt{R_{0}R_{1}}\right)\right\}. \end{array}$$

We then rewrite *W* using a sum-to-product formula and we then obtain

(A28) $$\begin{array}{c} W=2\sinh\left\{{\left|\alpha \right|}^{2}\left(1+R_{0}\right)\right\}\sinh\left\{-{\left|\alpha \right|}^{2}\left(1+R_{1}\right)\right\}+2\sinh^{2}\left\{{\left|\alpha \right|}^{2}\left(1+\sqrt{R_{0}R_{1}}\right)\right\}. \end{array}$$

The first term on the right-hand side (RHS) is negative, and the second term on the RHS is positive because $0\leq R_{0}<R_{1}\leq 1$. Furthermore, the absolute value of the first term is greater than that of the second term because $\left(1+R_{0}\right)\left(1+R_{1}\right)>{\left(1+\sqrt{R_{0}R_{1}}\right)}^{2}$, and sinh is odd and increases monotonically. Therefore, W<0, and thus B<0. □
